# Statistical Distance as a Measure of Physiological Dysregulation Is Largely Robust to Variation in Its Biomarker Composition

**DOI:** 10.1371/journal.pone.0122541

**Published:** 2015-04-13

**Authors:** Alan A. Cohen, Qing Li, Emmanuel Milot, Maxime Leroux, Samuel Faucher, Vincent Morissette-Thomas, Véronique Legault, Linda P. Fried, Luigi Ferrucci

**Affiliations:** 1 Groupe de recherche PRIMUS, Department of Family Medicine, University of Sherbrooke, 3001 12e Ave N, Sherbrooke, QC, J1H 5N4, Canada; 2 Department of Chemistry, Biochemistry and Physics, Université du Québec à Trois-Rivières, 3351, boul. des Forges, C.P. 500, Trois-Rivières, QC, G9A 5H7, Canada; 3 Economics Department, ESG, Université du Québec à Montréal, 315 rue Sainte-Catherine Est, Montréal, QC, H2X 3X2, Canada; 4 Mailman School of Public Health, Columbia University, 722 W. 168th Street, R1408, New York, NY, 10032, United States of America; 5 Translational Gerontology Branch, Longitudinal Studies Section, National Institute on Aging, National Institutes of Health, MedStar Harbor Hospital, 3001 S. Hanover Street, Baltimore, MD, 21225, United States of America; Moffitt Cancer Center, UNITED STATES

## Abstract

Physiological dysregulation may underlie aging and many chronic diseases, but is challenging to quantify because of the complexity of the underlying systems. Recently, we described a measure of physiological dysregulation, *D_M_*, that uses statistical distance to assess the degree to which an individual’s biomarker profile is normal versus aberrant. However, the sensitivity of *D_M_* to details of the calculation method has not yet been systematically assessed. In particular, the number and choice of biomarkers and the definition of the reference population (RP, the population used to define a “normal” profile) may be important. Here, we address this question by validating the method on 44 common clinical biomarkers from three longitudinal cohort studies and one cross-sectional survey. *D_M_*s calculated on different biomarker subsets show that while the signal of physiological dysregulation increases with the number of biomarkers included, the value of additional markers diminishes as more are added and inclusion of 10-15 is generally sufficient. As long as enough markers are included, individual markers have little effect on the final metric, and even *D_M_*s calculated from mutually exclusive groups of markers correlate with each other at *r*~0.4-0.5. We also used data subsets to generate thousands of combinations of study populations and RPs to address sensitivity to differences in age range, sex, race, data set, sample size, and their interactions. Results were largely consistent (but not identical) regardless of the choice of RP; however, the signal was generally clearer with a younger and healthier RP, and RPs too different from the study population performed poorly. Accordingly, biomarker and RP choice are not particularly important in most cases, but caution should be used across very different populations or for fine-scale analyses. Biologically, the lack of sensitivity to marker choice and better performance of younger, healthier RPs confirm an interpretation of *D_M_* physiological dysregulation and as an emergent property of a complex system.

## Introduction

While the fundamental biological mechanisms of aging are not yet clear, an increasing number of researchers are converging on the idea that aging is complex and multi-factorial [1,2], possibly emerging from a dysregulation of the physiological regulatory networks that maintain organismal homeostasis [3,4,5,6], also called allostatic load [7]. While this hypothesis is attractive, the complexity of the systems involved makes it hard to test it, and methods are needed to measure the relative stability of the system. A few studies have applied sophisticated statistical approaches with confirmatory but complex results [8,9,10,11,12]. The operationalization of multi-system dysregulation and allostatic load remains a challenge for the field [7].

Recently, we proposed a method for measuring physiological dysregulation based on the statistical distance (*D*_*M*_) among a set of biomarkers [13]. Statistical distance uses the correlation structure of the biomarkers to measure how aberrant each individual’s profile is with respect to the overall average (centroid) of the reference population. We hypothesized that individuals with a more deviant overall biomarker profile were more dysregulated; we validated this interpretation by demonstrating that *D*_*M*_ increases with age within individuals and, after controlling for age, predicts mortality, frailty onset, and chronic disease onset [13,14,15]. This is true despite the fact that *D*_*M*_ is often uncorrelated with its component biomarkers. Additionally, we showed that this was true for many different combinations among a limited set of 14 biomarkers that the signal increased as more biomarkers were included, and that the biomarkers need not be chosen based on specific *a priori* hypotheses regarding their role in aging. These findings, if generalizable to other biomarkers and contexts, have important biological implications: the ability to detect a similar signal with different combinations of markers, and to better detect it with more markers (regardless of which), would suggest that dysregulation is a diffuse property of overall system state rather than a function of a small number of physiological pathways.

However, two important aspects of validation remain to be completed. First, the signal of *D*_*M*_ is potentially confounded by or mixed with signals of dysregulation in particular systems or by the effect of specific biomarkers. Therefore, to validate the use of *D*_*M*_ for assessing general physiological dysregulation, we must quantify how *D*_*M*_ values calculated from many different sets of biomarkers correlate, using a larger pool of biomarkers than our previous studies. If the redundancy were very high, namely if *D*_*M*_ values calculated based on different sets of biomarkers are highly correlated, it would indicate that physiological dysregulation could be measured with virtually any set. On the other hand, if *D*_*M*_ values are little or un-correlated, this would indicate that physiological dysregulation is not approximated in the same way by different biomarker sets. In this case, increasing the number of biomarkers may be necessary to achieve a better signal of a general physiological dysregulation at the organism level. Second, in order to identify the degree to which a biomarker profile is deviant or aberrant, it is necessary to define “normal.” This is achieved through use of a reference population (RP), based on which the mean vector and variance-covariance structure of the variables is estimated. *D*_*M*_ measures the distance from this RP mean [16]. For most applications, *D*_*M*_ is calculated using the entire sample available as the RP. However, when using *D*_*M*_ as a measure of physiological dysregulation, it is not clear that the entire sample is the appropriate RP: for example, perhaps it is best to select a young, healthy sub-population from the whole population in order to best estimate parameters associated with a state of “robust health.” However, increased sample size for the RP should also be important to improve estimation of population parameters; if sample size is sufficiently important, it might be preferable to use the entire population rather than to try to choose healthy subsets. In turn, it might sometimes be advisable to use an outside population that is younger and/or has a larger sample size as an RP, despite potential differences in the population composition. Lastly, it is possible that population demographic composition (by sex, race, etc.) could influence the appropriateness of a population as a reference.

Addressing the questions outlined above would provide confidence in the use of *D*_*M*_ as a measure of physiological dysregulation and concrete guidance as to how to use it. They also could provide substantial biological insight into what physiological dysregulation is. For example, if *D*_*M*_ is highly robust to biomarker choice, it will suggest a diffuse signal of dysregulation and imply that dysregulation is fully a system-level property of a complex system (i.e., an integrated regulatory network). If demographic characteristics of RPs beyond age have little influence on the calculation of *D*_*M*_, this would imply that a healthy biomarker profile is very similar under different demographic contexts.

To address these issues, we investigated the consistency of the physiological dysregulation signal across *D*_*M*_ values from different biomarker sets (overlapping and non-overlapping) and numbers, by testing their correlations with each other and with age. We also performed a series of sensitivity analyses [17] designed to test the robustness of the performance of *D*_*M*_ when it is calculated based on various RPs. We assessed the performance of different versions of *D*_*M*_ based on their correlations with each other and their ability to predict mortality, frailty, chronic diseases, and changes with age within individuals. We replicated the analyses on data from three longitudinal cohort studies and one cross-sectional survey.

## Materials and Methods

### Data Sets

InCHIANTI (*Invecchiare in Chianti*) is a prospective study with participants randomly selected from two towns in the Chianti area in Italy (1156 adults aged 65–102 and 299 aged 20–64), described in detail elsewhere [18]. Baseline visits occurred in 1998–2000 with follow-ups in 2001–2003, 2005–2006 and 2007–2008. WHAS (Women’s Health and Aging Study) is a set of two complementary prospective studies of elderly women from Baltimore City and County in Maryland, USA [19,20]. WHAS I included 1002 women aged ≥65 among the one-third most disabled in their community. WHAS II included 436 women aged 70–79 among the two-thirds least disabled. Baseline visits occurred in 1992–95 and in 1994–96 for WHAS I and II, respectively, with follow-up visits conducted 1.5, 3, 6, 7.5, and 9 years later. BLSA (Baltimore Longitudinal Study of Aging) is longitudinal study of ageing that started in 1958 [21]. Participants were aged 21–96 and were largely middle- to upper-class, from the Baltimore and Washington DC area, and were followed approximately every two years. The study design was modified in 2003 whereby the number of biomarker measured increased substantially [21]. For this study, we are thus using data collected since 2003. NHANES (National Health and Nutrition Examination Survey) is a continuous cross-sectional stratified survey designed to be representative of the US population. Data are updated approximately every year and are made available freely (Centers for Disease Control and Prevention of the U.S. Department of Health and Human Services; http://www.cdc.gov/nchs/nhanes.htm). We used data from the waves 1999–2000, 2001–2002, 2003–2004, 2005–2006, 2007–2008, and 2009–2010, which have been described in detail elsewhere [22].

All aspects of WHAS, InCHIANTI, and BLSA research were approved by the ethics committees at the institutions responsible for data collection, and this secondary analysis was approved by the ethics committee (*Comité d’éthique de la recherche en santé chez l’humain*) at the *Centre de recherche clinique du CHUS*, project # 11–020. Participants signed informed consent for data collection and analysis. Although the data used in these analyses cannot be freely shared due to confidentiality constraints related to human medical data, they are all available to researchers submitting an appropriate research proposal: WHAS at https://jhpeppercenter.jhmi.edu/ec_proposal/login.aspx, InCHIANTI at http://www.inchiantistudy.net/obtain_data.html, and BLSA at http://www.blsa.nih.gov/researchers.

### Biomarker Selection

**Fig 1:**
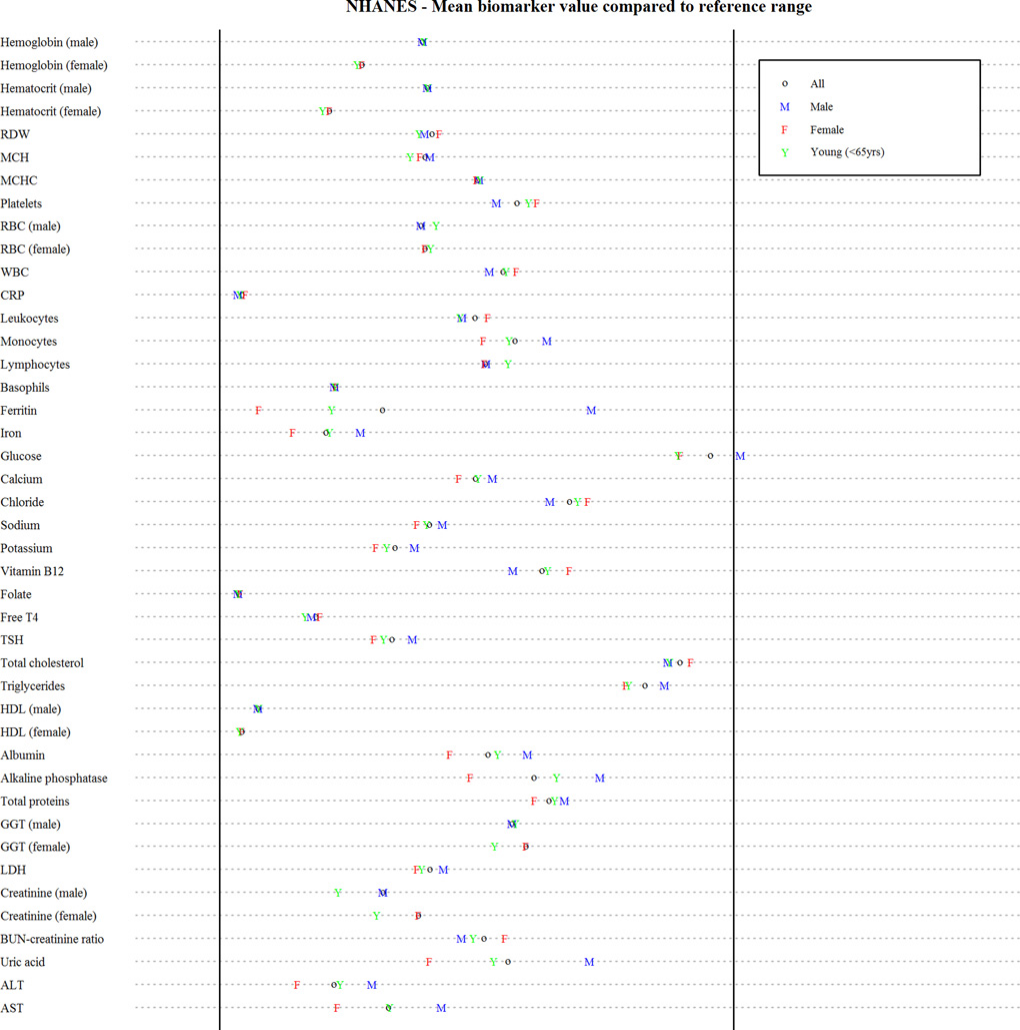
Mean biomarker values for NHANES in relation to reported reference ranges. Mean values for each biomarker were normalized according to the reported minimal and maximal normal values, represented by the vertical lines. For biomarker with only one specified normal value, the other vertical line represents minimal or maximal value for the data set (see S1 Table for details). Graphs for other data sets can be found in S1–S3 Figs.

For analyses on the sensitivity of *D*_*M*_ to which biomarkers are included, we selected 44 biomarkers that were available in multiple studies with large sample sizes (>1,000 observations per data set). Due to data availability, respectively one and nine markers were excluded from WHAS and NHANES (Table 1). This resulted in a final list that was composed nearly exclusively of markers that are commonly used in clinic. Fig 1 shows the mean values for each biomarker in NHANES and by subset, in relation to reported reference ranges (see S1 Table for details and S1–S3 Figs for graphs for other data sets). In other data sets, mean values for some biomarkers (e.g. lactate dehydrogenase, total cholesterol, glucose) lie outside reported ranges, which is to be expected with an overrepresentation of older adults. The raw correlations between all biomarkers are shown in S4 through S7 Figs; overall, they are similar from one database to the other (Figs were drawn with the corrplot package for R).

**Table 1:** Biomarkers used in this study and number of observations for each data set.

****Biomarkers****	****BLSA****	****WHAS****	****InCHIANTI****	****NHANES****
A/G ratio	2975	2824	3637	n.a.
Albumin (serum)	2977	3736	3637	39828
Alkaline phosphatase	2977	3725	3640	39825
ALT	2963	3738	3648	39734
AST	2977	3737	3646	39732
Basophil %	2928	2566	3641	51193
BUN/creatinine ratio	2410	3734	3647	n.a.
Calcium	2977	3721	3637	39826
Cholesterol	2976	3030	3649	46625
Chloride	2977	3726	3638	39818
Serum creatinine	2977	3737	3651	n.a.
C-reactive protein	1349	2748	3627	47982
DHEAS	2061	2980	2991	n.a.
Eosinophil %	2954	2566	3641	n.a.
Estradiol	1908	2726	1927	n.a.
Ferritin	2965	2827	3617	25705
Folate (serum)	2943	2807	2166	49787
Free T4	2928	n.a.	1199	8630
GGT	2854	2826	3644	39823
Glucose	2969	3738	3648	16095
Hemoglobin	2953	3641	3643	51335
Hematocrit	2956	3641	3643	51335
HDL	2976	3267	3646	46619
IGF-1	2283	2784	2964	n.a.
IL-6	1342	2814	2974	n.a.
Iron	2881	2813	3637	39810
Potassium	2973	3720	3643	39823
LDH	2948	2810	3634	39730
Lymphocyte %	2955	2566	3641	51193
MCH	2953	3641	3643	51335
MCHC	2953	3641	3643	51335
Magnesium	2973	2778	3635	n.a.
Monocyte %	2954	2566	3641	51193
Neutrophil %	2955	2565	3642	51193
Platelets	2954	3620	3643	42535
Red blood cell count	2928	3641	3643	42536
RDW	2926	3640	3643	51335
Sodium	2977	3726	3644	39825
Total protein	2951	3739	3641	39789
Triglycerides	2947	3029	3649	21000
TSH	2927	2998	1153	4392
Uric acid	2944	2823	3625	39822
Vitamin B12	2940	2803	2166	32776
White blood cells	2930	3641	3643	51332

### Mahalanobis Distance Calculation

Once combinations of biomarkers were selected (see below), *D*_*M*_ was calculated as previously described [13] using the mahalanobis function in R. For analyses on sensitivity of *D*_*M*_ to biomarker selection, we used all observations as the RP to compute *μ* (the vector of mean biomarker values) and **S** (the variance-covariance matrix among the biomarkers). *D*_*M*_ was log-transformed for subsequent analyses. For analyses on RP, we distinguish the “study population” (the individuals for whom we calculate *D*_*M*_), from the RP, the individuals based on whom we calculate *μ* and **S**. For the three longitudinal studies (WHAS, InCHIANTI, BLSA), we used all visits available for an individual, i.e. those for which we had measurements for all biomarkers. Thus, one visit for one person equated to one observation. For NHANES only one visit per individual was available. All statistical analyses were conducted in R v3.0.1 and code is available upon request.

### Analyses for Sensitivity of D_M_ to Biomarker Composition

For analyses on sensitivity of *D*_*M*_ to biomarker composition, the goal was to evaluate (a) the optimal/minimum number of biomarkers to include in *D*_*M*_; (b) which biomarkers to include or exclude, if there were major differences; and (c) the extent to which *D*_*M*_ produces a robust signal independent of biomarker composition, indicating that it detects a system-level property. However, in order to evaluate the performance of different combinations of biomarkers, we need something to compare them to. We chose to make two separate comparisons. First, we compared the signal of one random combination to another random combination using Pearson correlation coefficients. In this way, we could identify how best to measure a general signal of *D*_*M*_, i.e. one that does not depend too much on what markers are included (see below). Second, we used age as an external benchmark. Each of these analyses is described in detail below.

#### Correlation among D_M_S calculated using random, mutually exclusive pairs of biomarkers

*D*_*M*_ was generated using random, mutually exclusive combinations of biomarkers so as to be able to study how the number of biomarkers included (*N*_*bm*_) and the identity of the biomarkers included influenced the stability of the *D*_*M*_ signal. It was not computationally feasible to study all possible biomarker combinations (reaching a maximum of 10^19^ possibilities with 15 biomarkers per group), so for each *N*_*bm*_ in 2 ≤ *N*_*bm*_ ≤ 22 we generated 5000 random combinations by sampling the 44 markers without replacement. In each case (5000 × 21 levels of *N*_*bm*_), we then generated a paired, non-overlapping combination containing the same number of markers selected from among those not included in the initial combination. This allowed us to compare the performance of different versions of *D*_*M*_ where the biomarkers are mutually exclusive but *N*_*bm*_ is equal. In particular, we could assess how strongly alternative versions of *D*_*M*_ correlated with each other, removing any redundancy due to shared biomarker composition. Note that, while it was essential that paired combinations be mutually exclusive, this restricted the maximum *N*_*bm*_ to 22 of the 44 markers. Also, since we were more interested in the distribution of correlations than in testing the significance of each one, we did not control for the non-independence of observations coming from the same individuals. However, we repeated the analyses using a single randomly selected visit per individual to insure that non-independence did not bias our conclusions in analyses using all observations.

By storing the information about which biomarkers were in each combination, we could assess the association between the 5000 × 21 correlation coefficients and whether or not each marker was included in one of the two groups, as well as between the correlation coefficient and *N*_*bm*_. To do this, we ran linear regression to examine the association between the correlation coefficient (the dependent variable) and either *N*_*bm*_ or the presence/absence of each biomarker in the combination (the independent variable(s)). While we do not interpret each correlation in terms of significance (as aforementioned), we used the *p*-values of the Pearson correlation to filter out “insignificant” correlations (p>0.05) in order to reduce the noise for the linear regressions, as low correlations are more likely to be truly insignificant, hence non-informative about the effect of a given biomarker.

#### Association between age and D_M_S calculated using random biomarker combinations

In order to assess how *N*_*bm*_ and biomarker choice affected the association between *D*_*M*_ and age, we used 5000 random combinations of biomarkers for each *N*_*bm*_ in 2 ≤ *N*_*bm*_ ≤ 44, this time without pairing or mutual exclusivity. The relationship of *D*_*M*_ with age is non-linear, and in particular there are conflicting effects of within-individual increases with age and higher mortality rates among individuals with higher *D*_*M*_ [13,15]. Accordingly, the correlation of *D*_*M*_ with age is not very informative, and a more sophisticated measure of association was needed. Hence, for each combination (5000 × 43 levels of *N*_*bm*_) we regressed log-transformed, standardized, *D*_*M*_ values on age by fitting linear and quadratic age terms, and extracted the multiple R-squared from the model, generating a measure of the variance in age explained by *D*_*M*_. In this way, we could use linear regression to examine the association between the multiple R-squared (the dependent variable, a measure of the association between *D*_*M*_ and age) and either *N*_*bm*_ or the presence/absence of each biomarker in the combination (the independent variable(s)).

### Analyses for Sensitivity of D_M_ to RP Characteristics

For sensitivity analyses on RPs, we used 12 biomarkers that were selected for our original study [13] as results from the biomarker choice analyses suggested that inclusion of 10–15 markers is generally sufficient for a good signal. The markers used were hemoglobin, hematocrit, red blood cell counts (RBC), sodium, calcium, potassium, chloride, cholesterol, creatinine, the blood-urea nitrogen (BUN) to creatinine ratio, albumin, and basophil percentage among white blood cells. The BLSA data set was not used for analyses on RPs due to (a) the lack of outcome data such as in WHAS and InCHIANTI, and (b) the lack of a large population of younger adults, such as in NHANES.

To evaluate the effect of different RPs, *D*_*M*_ was analysed in relation to age, mortality, frailty, cardiovascular disease (CVD) and number of comorbidities, with RPs that produce stronger associations with these variables presumed to be “better.” NHANES has no longitudinal data so only the correlation between *D*_*M*_ and age is presented. For InCHIANTI and WHAS, individual changes in log-*D*_*M*_ with age were modelled using linear regression models for each individual to estimate his/her slope; weighted *t*-tests were then used to assess whether the slope was significantly different from zero, weighted by the number of observations per individual. This method, while theoretically slightly inferior to a full multi-level model, was much more computationally feasible for the large number of analyses we were running. The relationship between *D*_*M*_ and subsequent mortality was modelled using Cox proportional hazards models (coxph function, survival package), controlling for a spline of age. Frailty was measured as the number of Fried’s frailty criteria present (0–5) and assessed using linear regression controlling for age, as was number of comorbidities [23]. CVD was assessed using regression controlling for age, but differently in WHAS and InCHIANTI based on data constraints (see S1 Text for details).

The above analyses were run for a large variety of combinations of RP and study population. The key parameters that were varied were (a) data set; (b) sample size of 50, 100, 200, 300 or full population, using random sub-samples (only pertinent for the RP); (c) age range (only applied to the RP); (d) sex (NHANES and InCHIANTI only); and (e) race (WHAS only). Each parameter combination could be applied to either the study population or the RP; for example, we could examine the performance of RP that was from NHANES, sample size 200, aged 20–40, male, and black for calculating values in a study population that was from InCHIANTI, aged 65+, and female. However, the number of possible such combinations far exceeded our analytical capacity; accordingly, we manually chose the most pertinent combinations, generally assessing one parameter “axis” at a time, and occasionally looking at their interactions. Each sensitivity analysis for a given RP-study population pair was conducted in replicate on 100 randomly chosen combinations among the 4095 possible combinations of the 12 biomarkers. Each sensitivity analysis is thus expressed as a summary of the predictive power across the 100 combinations, as described below.

In addition, we performed a series of meta-regressions to test the importance of RP characteristics across the many different RP-study population combinations modelled. For each combination of RP-study population-outcome, we calculated the percentage of the 100 models (i.e., 100 biomarker combinations) that was significant at α = 0.05. This percentage was used as the dependent variable in meta-regressions, and the independent variables were various combinations of health outcome (age slope, mortality, frailty, etc.) and RP or study population traits such as sex, age, race, their interactions, etc. as appropriate.

#### Graphical representation of RP results

Because of the large number of analyses to be presented, we developed a graphical summary method using matrices of filled, shaded rectangles. Each rectangle simultaneously summarizes the effect size, *p*-value, and percent of significant *p*-values (at α = 0.05) among the 100 analyses. The percentage of significant *p*-values is represented by the height of shading within the rectangle: white represents no significant result, all shaded indicates that all 100 analyses were significant. The colour of the shading represents the direction of the effect (blue is a positive effect, red a negative effect), and the hue represents the average *p*-value among the significant p-values, with darker hues indicating lower *p*-values (greater significance). Each matrix of rectangles has a row for each possible outcome (age, mortality, CVD, etc.) and a column for each different RP. The leftmost column is a “reference RP,” i.e., a relatively straightforward choice, such as using the entire study population as its own RP. The other columns are compared to this choice, with the width of the rectangle representing the average effect size among significant analyses, relative to the effect size of the rectangle in the leftmost column and the same row. Wider rectangles indicate larger effect sizes. Accordingly, all rectangles in the leftmost column have the same width, and the width of other rectangles can only be compared to rectangles in the same row. While the details of the interpretation of these Figs are thus complex, the visual result is simple: more and darker blue means better performance.

## Results

### Pairwise D_M_ Correlations and Predictive Value of Age for Different Sets of Biomarkers

**Fig 2:**
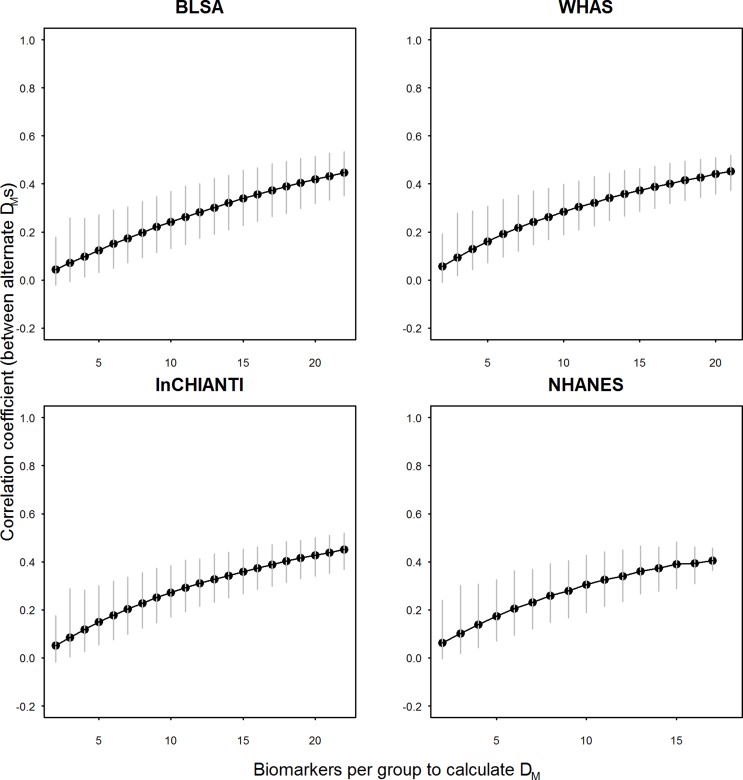
Mean correlation between pairwise *D*_*M*_ values as a function of biomarker number. Grey vertical bars indicate 2.5 to 97.5 percentiles of observed correlation coefficients calculated between ~5,000 random mutually exclusive pairs generated from a pool of 44 markers.

The correlation between pairs of *D*_*M*_ was always positive and increased with the number of biomarkers per group. The patterns obtained for the different data sets were remarkably similar: approaching 20 markers per group, the correlation starts to level off at around 0.4 in all four data sets, with limited variation around the mean as shown by the 2.5 to 97.5 percentiles of observed correlation coefficients (Fig 2). However, whether a plateau truly occurs at around 20 biomarkers is not clear since our study did not go beyond 22 biomarkers per group (half of the 44 available, to preserve mutual exclusivity). The results obtained with the full data sets vs. the data sets restricted to one visit per individual were similar (not shown). Therefore, hereafter we only present the results for the former.

**Fig 3:**
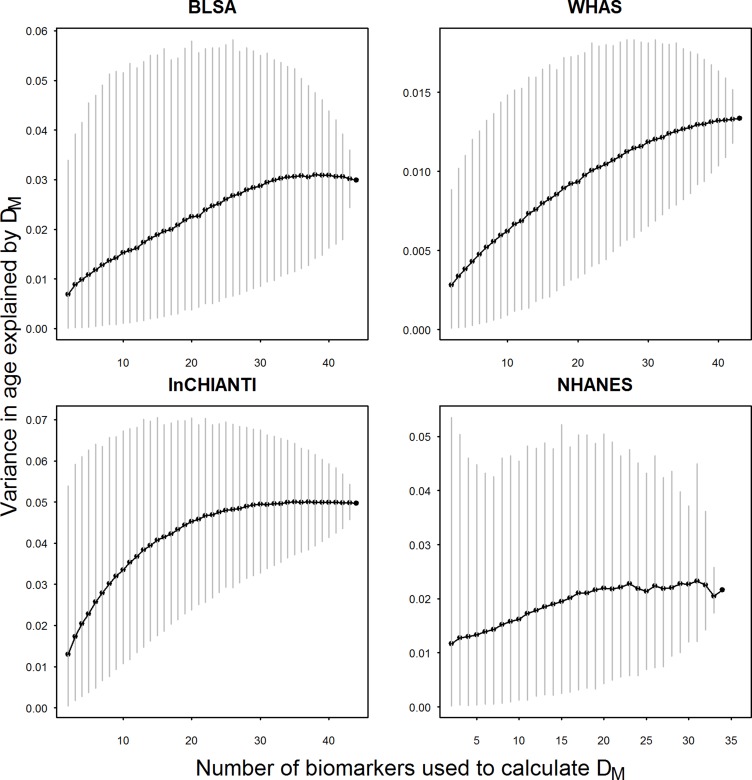
Mean variance of predicted *D*_*M*_ values with age as a function of biomarker number. Grey vertical bars indicate 2.5 to 97.5 percentiles of observed variances in age explained by *D*_*M*_ calculated from ~5,000 random combinations generated from a pool of 44 markers.

The relationship between *D*_*M*_ and age is somewhat less stable across data sets than the correlations. Overall, the variance explained by quadratic regressions of predicted *D*_*M*_ with age tends to increase when more biomarkers are included in *D*_*M*_ calculation, but reaches a plateau at around 30 biomarkers (Fig 3). Note that we could show all 44 markers in Fig 3 because we were not constrained to use mutually exclusive groups, as in Fig 2. This global pattern is true for BLSA, InCHIANTI and WHAS but not NHANES, which is the only cross-sectional study. The larger error bars indicate greater heterogeneity in variance explained across biomarker combinations.

### Contribution of Individual Biomarkers to pairwise D_M_ Correlations and Relation to Age

**Fig 4:**
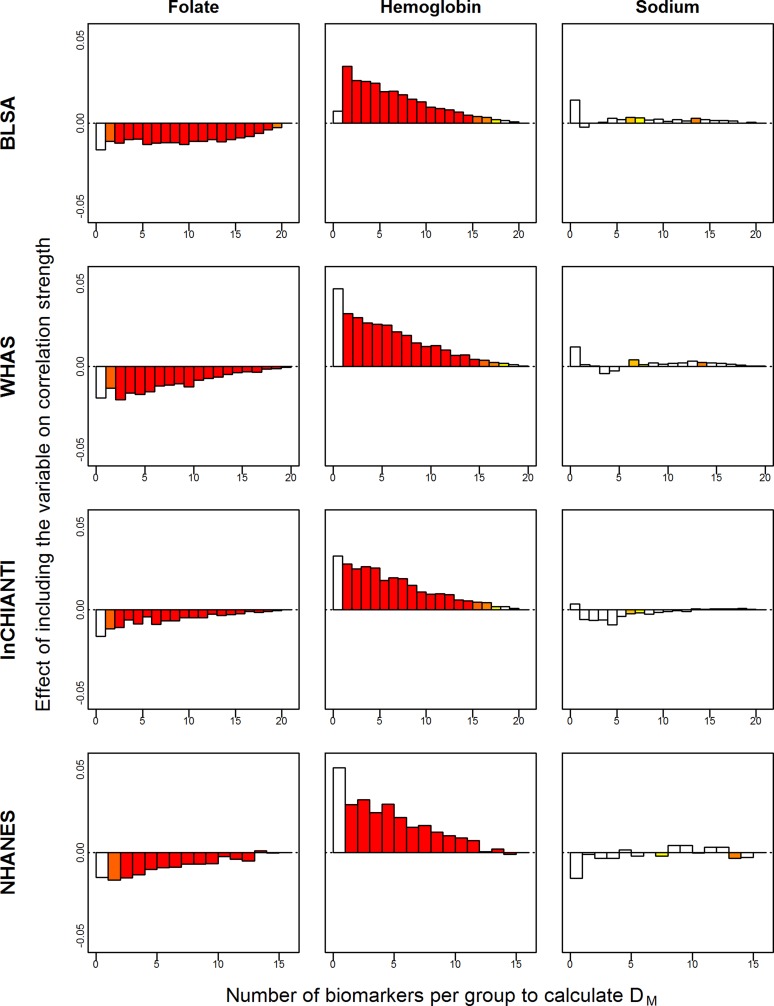
Contribution of selected individual biomarkers to pairwise *D*_*M*_ correlations, as a function of biomarker number. The X-axis represents the number of biomarkers per group (*N*_*bm*_) and the Y-axis reports the coefficient (*β*) from a linear regression of the *D*_*M*_ pairwise correlations on *N*_*bm*_. *βs* represent the deviation from the average correlation when a given biomarker is included in the calculation of *D*_*M*_; positive values thus indicate improved performance of *D*_*M*_, and negative values decreased performance. Colors indicate the magnitude of *p*-values, with darker red being more significant and white not significant. Graphs for all biomarkers can be found in S8–S11 Figs.

Individual biomarkers contributed in diverse ways to the correlation between *D*_*M*_ values, following three major patterns: those with a positive effect, i.e. increasing the strength of the correlation; those with a negative effect, i.e. decreasing the strength of the correlation; those with no clear effect in either direction. Selected examples of biomarkers showing these three patterns are illustrated in Fig 4 and graphs for all biomarkers can be found in S8–S11 Figs. Two patterns emerge from this analysis. First, whether positive or negative, the effect of a marker on the strength of the correlation decays with increasing *N*_*bm*_ and typically becomes negligible at highest *N*_*bm*_ values. Second, the effect of specific markers is quite consistent from one data set to the other (Fig 4, S8–S11 Figs): markers that have a strong positive (e.g. hemoglobin, MCH, neutrophils) or negative (e.g. basophil, folate, vitamin B12) effect tend do so in all data sets, while those having a weak effect in one set tend to have either a similar or non-significant effect in other sets (e.g., HDL, iron, sodium). Notably, several blood markers follow the same pattern, with a strong positive effects on the strength of the correlation that declines sharply with increasing *N*_*bm*_ (hemoglobin, haematocrit, RBC and to a lesser extent red blood cell width; RDW). A few markers depart from these general rules. For example, alanine aminotransferase, estradiol and free thyroxine (T4) clearly show a positive effect on the correlation in some data sets and a negative effect in others (S8–S11 Figs).

**Fig 5:**
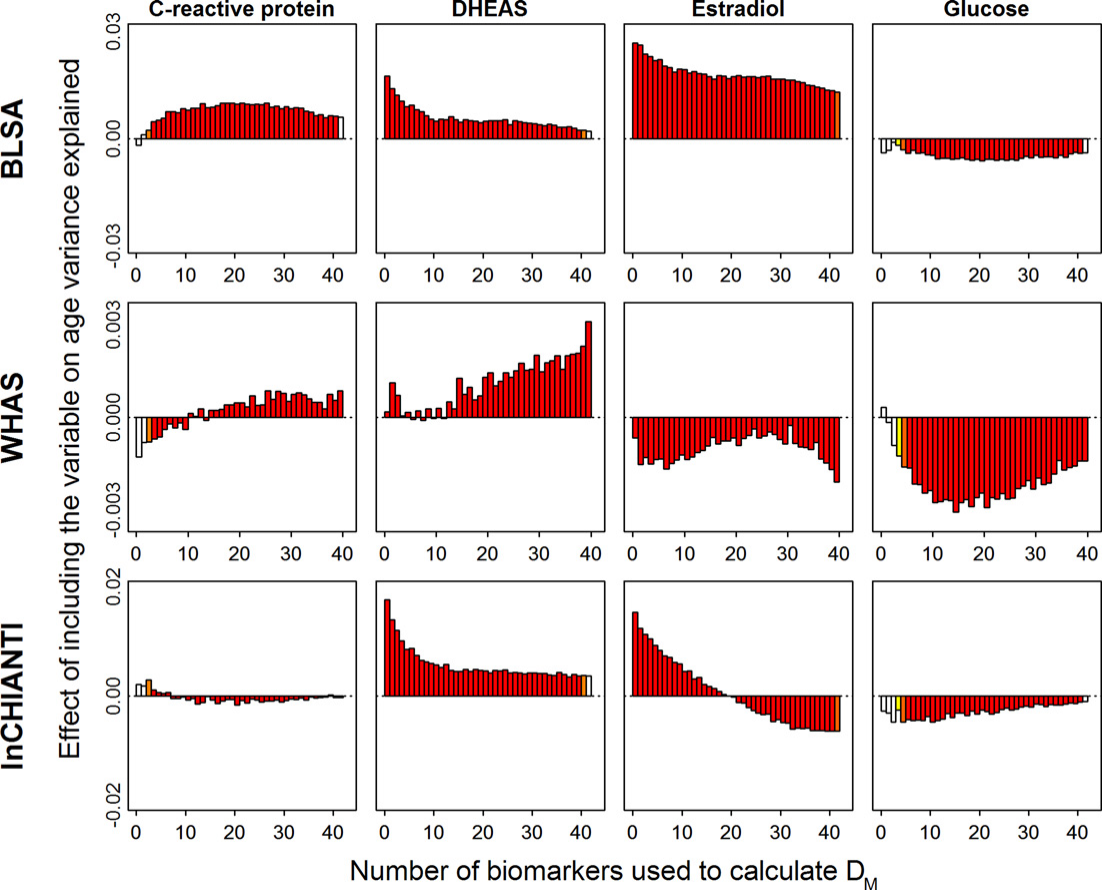
Contribution of selected individual biomarkers to change in variance in age explained by *D*_*M*_. The X-axis represents the number of biomarkers per group (*N*_*bm*_), while the Y-axis reports the change in how much variance in age is predicted by *D*_*M*_ with the inclusion of the given biomarker, based on a meta-regression of all the R-squareds calculated for individual quadratic regressions of age and *D*_*M*_. Colors indicate the magnitude of *p*-values, with darker red being more significant and white not significant. Graphs for all biomarkers can be found in S12–S15 Figs.

The effects of including individual biomarkers on the association of *D*_*M*_ with age were much less clear. Several examples are shown in Fig 5, with full results in S12–S15 Figs. Results differed across data sets in most cases, often dramatically. For example, CRP has a large positive effect in BLSA, an effect that goes from clearly negative to clearly positive as *N*_*bm*_ increases in WHAS, and no major effect in InCHIANTI. The smaller y-axis scale for WHAS is probably due to less variance in age explained by *D*_*M*_ in this data set due to the smaller age range of participants. For correlations, the effect of individual markers consistently decreased as *N*_*bm*_ increased, but for the association of *D*_*M*_ with age, many patterns were observed: stable positive effects, stable negative effects, effects that go from negative to positive and vice-versa, effects that are non-linearly associated with *N*_*bm*_ such that intermediate values of *N*_*bm*_ are either higher or lower than extreme values, etc. In short, the inclusion or exclusion of individual variables in *D*_*M*_ appears to be much more important for its association with age than for correlations among alternative versions of *D*_*M*_. However, the details of these effects appear to depend on many other factors.

### Sensitivity of D_M_ Analyses to RP Choice

**Fig 6:**
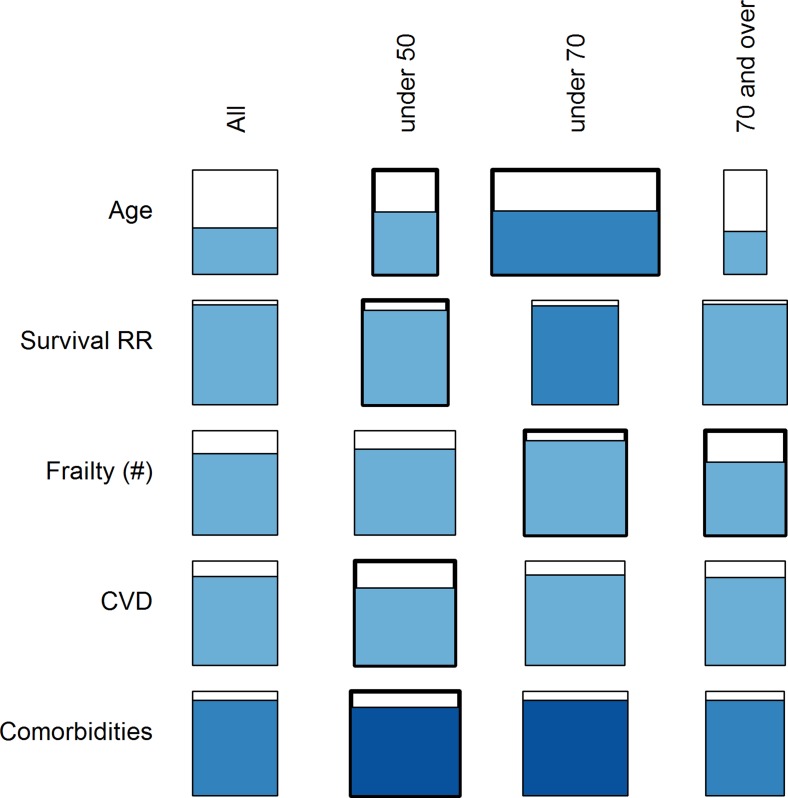
Effects of RP age intervals on prediction of age and health outcomes. The study population represented here is the full InCHIANTI data set with InCHIANTI RPs covering different age intervals. The width of the rectangle represents the average effect size among significant analyses, relative to the effect size of the rectangle in the leftmost column (entire study population as its own RP). The percentage of significant p-values is represented by the height of shading within the rectangle, the shading colour represents the direction of the effect (blue is a positive effect), and the hue represents the average *p*-value among the significant p-values, with darker hues indicating lower *p*-values.

**Fig 7:**
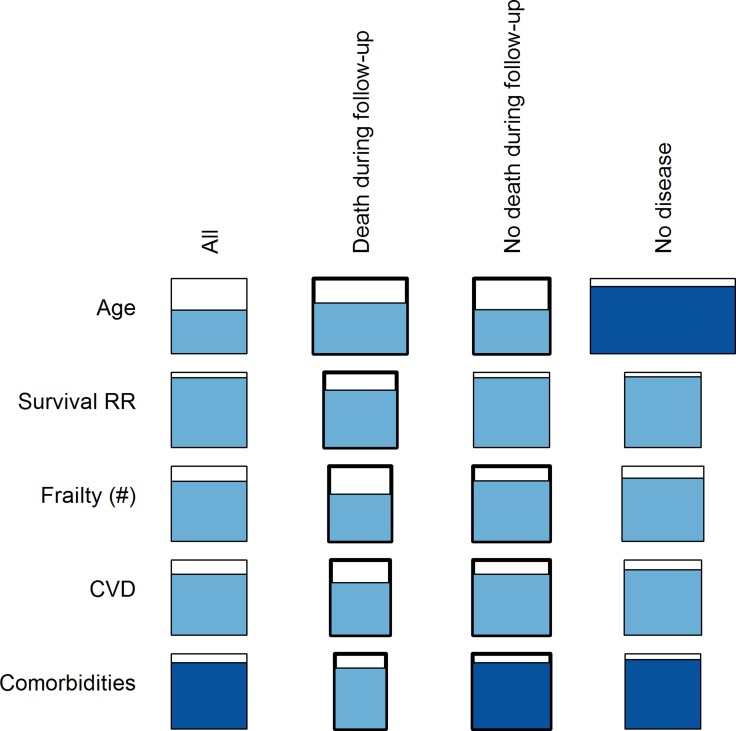
Effects of RPs’ survival and health status on prediction of age and health outcomes. The study population represented here is the full InCHIANTI data set with InCHIANTI RPs defined according to survival and health status. The width of the rectangle represents the average effect size among significant analyses, relative to the effect size of the rectangle in the leftmost column (entire study population as its own RP). The percentage of significant p-values is represented by the height of shading within the rectangle, the shading colour represents the direction of the effect (blue is a positive effect), and the hue represents the average *p*-value among the significant p-values, with darker hues indicating lower *p*-values.

**Fig 8:**
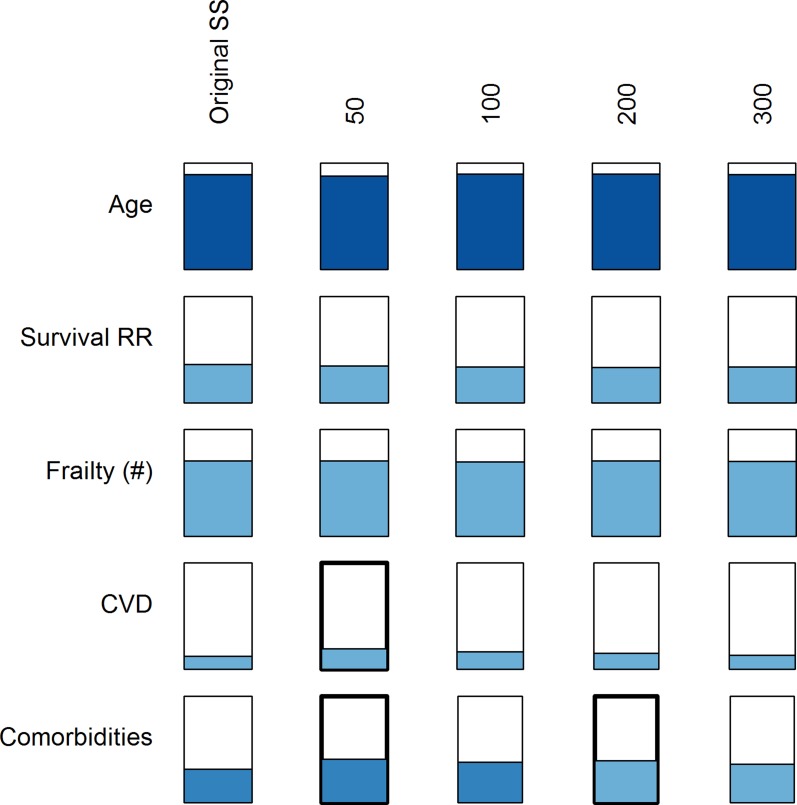
Effects of RP sample size on prediction of age and health outcomes. The study population represented here are individuals aged 20–70 from the InCHIANTI data set with RPs of various sample sizes drawn randomly from the study population. The width of the rectangle represents the average effect size among significant analyses, relative to the effect size of the rectangle in the leftmost column (entire study population as its own RP). The percentage of significant p-values is represented by the height of shading within the rectangle, the shading colour represents the direction of the effect (blue is a positive effect), and the hue represents the average *p*-value among the significant p-values, with darker hues indicating lower *p*-values.

There was a clear tendency for better performance using younger RPs, especially for a positive slope of *D*_*M*_ with age (Fig 6 and S16 Fig). For example, in a regression analysis looking only at InCHIANTI as both RP and study population, using an RP of patients aged 20–50 or 20–70 (as opposed to the whole population) improved model performance substantially (12% and 16%, p = 0.03 and 0.0004, respectively; Fig 6). Likewise, the use of healthier RPs (i.e., not dying or without comorbidities) for InCHIANTI clearly increased the average effect size and *p*-value for the slope of *D*_*M*_ with age, compared to the full data set (Fig 7). The only exception was the slope of *D*_*M*_ with age for those not dying during follow-up, probably due to a difference in the age composition of the two sub-populations. On the other hand, there was essentially no effect of sample size on the results (Fig 8). This was true in InCHIANTI, WHAS, and NHANES, both visually and using regression analyses. Sample size was never a significant explanatory variable in regression models.

**Fig 9:**
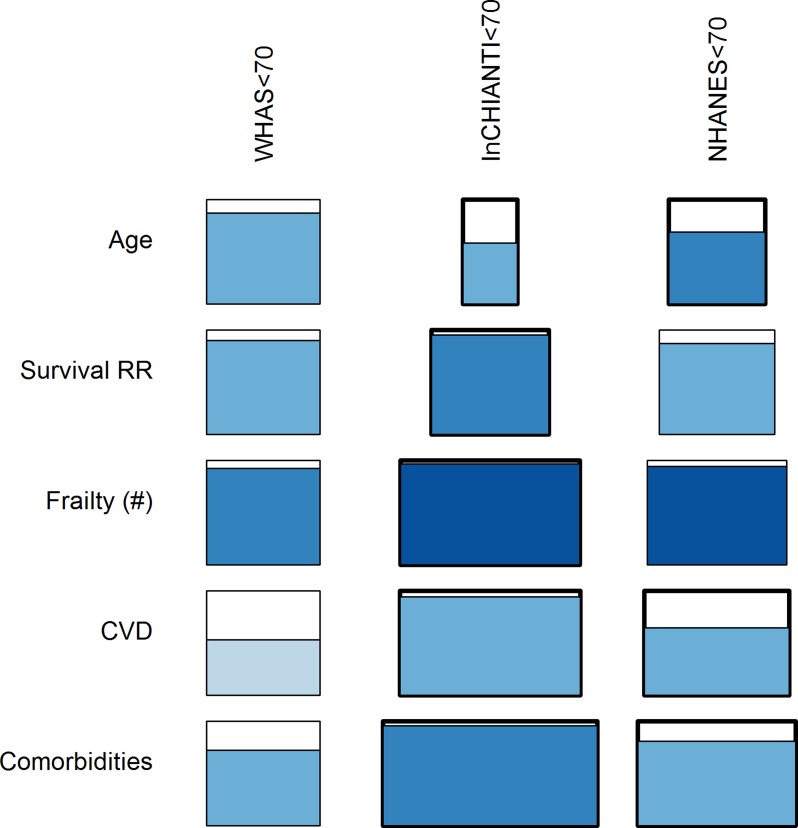
Effects of RP drawn from external young populations on prediction of age and health outcomes. The study population represented here is the full WHAS data set with young RPs from each of the three data sets as indicated. The width of the rectangle represents the average effect size among significant analyses, relative to the effect size of the rectangle in the leftmost column (entire study population as its own RP). The percentage of significant p-values is represented by the height of shading within the rectangle, the shading colour represents the direction of the effect (blue is a positive effect), and the hue represents the average *p*-value among the significant p-values, with darker hues indicating lower *p*-values.

The effects of population choice, as well as sex and race, are less clear but tend to demonstrate some sensitivity of model performance to RP variation (Fig 9 and S17–S19 Figs). The number of significant models among the 100 varied substantially depending on which data set was used for the RP and the study population. For example, WHAS performed substantially worse as its own RP (22% worse than InCHIANTI, p<0.0001; Fig 9), whereas using InCHIANTI as the study population, there was a substantial decrease in performance using WHAS or NHANES as RP, rather than InCHIANTI itself (-10% and -5%, p = 0.04 and 0.01, respectively; S17 Fig). Likewise, results were often markedly different using black, white, and mixed RPs in WHAS (S18 Fig). Qualitatively, conclusions went in the same direction, but the number of significant models, significance level, and effect size often differed substantially. Strangely, there were often opposing effects for effect size and significance, perhaps suggesting that results obtained for race are an artefact and should not be over-interpreted.

The sex of the RP and study population generally produced modest but significant effects on the results, based largely on analyses within InCHIANTI (S19 Fig). As with most analyses above, use of a different RP never changed overall conclusions, but the number of significant models and effect sizes varied a bit. Unlike for race, variation in number of significant models, significance level, and effect size were consistent with each other. The more consistent results than for race suggest that the sex composition of the RP may have real if modest effects on results.

## Discussion

This study assessed the sensitivity of *D*_*M*_ to biomarker choice and demographic composition of the RP, with the dual goals of establishing a standard methodology to calculate *D*_*M*_ and understanding the biological implications of its stability profile. Overall, we found that performance of *D*_*M*_ as a marker of physiological dysregulation increases with the inclusion of more biomarkers, but that there are diminishing returns at higher numbers of markers, and that 10–15 markers is generally sufficient to recover a strong signal. The choice of markers has relatively little effect on the correlations between different versions of *D*_*M*_, but can be important in more specific applications, such as measuring the strength of the association between *D*_*M*_ and age. However, which biomarkers improve *D*_*M*_ signal appears to be context-dependent, making it difficult to generate a list of preferred markers without a more extensive analysis across different populations. The effect of RP choice was also of moderate importance in some contexts: it appears generally better to use a younger and healthier RP, and one that is otherwise demographically similar to the study population. However, RP sample size does not matter much beyond 50.

These results are nuanced and complex rather than black-and-white, so we work through some of these details below; the overall take-home messages are: (a) We confirmed a general insensitivity of *D*_*M*_ to biomarker choice across 44 markers (compared to the 14 previously analysed), strengthening the conclusion that physiological dysregulation is an emergent property of system state not particularly linked to any single molecule, pathway, or physiological system. (b) A modest sensitivity of *D*_*M*_ to RP choice suggests that there is not a single universal optimal physiological state across populations, but that any differences are modest. (c) For most studies for most purposes, choice of biomarkers and RP will not have major impacts on the results as long as 10–15 relatively diverse markers are chosen and the RP is not too different from the study population. However, studies making fine-scale inferences should use caution and attempt to validate these choices.

Having a robust, simple, effective measure of physiological dysregulation would represent a major step in a number of fields. Dysregulation could serve as a proxy for individual health status in large-scale population surveys in fields such as demography, economics, and sociology, and may represent a substantial improvement over self-reported health, single-biomarker measures, or summed indices of criteria [24,25,26,27,28]. Clinically, a marker of dysregulation may improve on single biomarkers in certain contexts. For example, it may help predict impending frailty [15] or serve as a physiotype for frailty. In aging studies, dysregulation may serve as an approximation of biological age [29]. We recently showed that *D*_*M*_ works as a measure of body condition in wild animals [30], suggesting major applications in ecology as well, where existing measures of body condition have been criticized [31]. In studies using lab animals, *D*_*M*_ may serve as a simple measure of health status. In clinical trials, lack of ability to measure long-term outcomes is a major problem; *D*_*M*_ could be added to such trials as a secondary outcome to predict long-term benefits or harms of medications or other treatments. The implications of a robust measure of physiological dysregulation are thus wide-ranging.

### Biological implications

The better performance of younger and healthier RPs confirms a prediction and thus supports the interpretation of *D*_*M*_ as a measure of physiological dysregulation. Including sicker or older individuals in the RP will pull the RP mean away from an ideally healthy state to the extent that there are general differences in biomarker levels between young and old, healthy and sick. If *D*_*M*_ truly measures dysregulation, it is thus expected that distance from the mean of young, healthy individuals will provide a stronger signal than distance from the overall mean. The lack of strong sensitivity to sample size, sex, and data set confirms the idea of a generalized underlying signal, supporting the hypothesis of dysregulation and concordant with studies on allostatic load and aging [9,10,11,32,33,34,35]. On the other hand, the occasional sensitivity to RP parameter combinations and the effects of some biomarkers on the association between *D*_*M*_ and age suggests that physiological dysregulation does not proceed in a completely uniform fashion such that all biomarkers measure it interchangeably in all populations; likely there is some heterogeneity in dysregulation processes across biological sub-systems, in ways that may differ across populations.

An interesting parallel with our findings is a common measure of clinical frailty, the frailty index (FI). The FI is based on the accumulation of deficits during aging, and is expressed as a percentage of deficits observed among those assessed [36,37,38]. As with *D*_*M*_, FI shows minimal sensitivity to the choice of deficits, though the signal increases asymptotically as more deficits are added to the measure [39]. It would thus appear that both clinical deficits and biomarker dysregulation follow a similar pattern of detecting an underlying signal that is physiologically generalizable. We hypothesize that *D*_*M*_ may be a physiological equivalent to FI, and a precursor to other frailty measures such as Fried’s frailty phenotype [23]. Indeed, a recent study on biomarkers and FI shows that their inclusion in FI is highly concordant with a general FI signal [40]. The relationship between *D*_*M*_ and FI will be explored in future studies.

### Detailed methodological considerations

One of our more puzzling results was the important but inconsistent effects of biomarker choice on association with age. For example, why would including estradiol in *D*_*M*_ strengthen the association with age in BLSA, decrease it in WHAS, and improve it in InCHIANTI for small numbers of biomarkers but decrease it for large numbers of biomarkers (Fig 5)? There are probably two key answers to such questions. First, the demographics of the population are quite important. Our study populations differ markedly in composition by age, sex, race, and socio-economic status. It is evident that the small variance in age explained by *D*_*M*_ in WHAS is due to the limited age-range in that study. How estradiol affects the association of *D*_*M*_ with age in WHAS is a function of how it changes between ages ~65–90 in women, whereas how it affects the association of *D*_*M*_ with age in other populations depends also on its changes in men, and in younger women (i.e., pre-menopausal). Estradiol is an extreme example in this case, with major known differences in levels and changes between men and women, and pre-vs. post-menopause in women. Second, there are likely interactions with the other markers present. Two redundant markers that improve the association of *D*_*M*_ with age may each be quite important with smaller numbers of markers, but may decrease in importance with larger numbers of markers, as the probability increases for the other to be included.

As for the sensitivity of *D*_*M*_ analyses to RP choice, the results presented here simultaneously provide a clear and a complex picture. Generally speaking, most conclusions are unlikely to change as a function of RP choice. There was minimal sensitivity to sample size, indicating that 50 observations provide a robust estimate of the variance-covariance matrix. Unsurprisingly, the use of a younger or healthier RP significantly improved the model performance. At the same time, the details provide a much more complex picture. For instance, mixed results were obtained when the RP came from a different data set on a different continent. The different demographic characteristics between data sets make it hard to evaluate if this was due to demographic aspects versus other more specific population traits such as population-specific physiological profiles. In particular, the fact that WHAS contains only women 65 years and older made it impossible to compare the use of a young, two-gendered WHAS population as a reference. Also, small differences were observed depending on the sex of the RP and study population, but these effects were minor in terms of overall conclusions.

In contrast, using RPs that were racially distinct had a major impact on the results in WHAS, the only data set in which we could perform this analysis. We do not believe this finding is attributable to racially fixed differences in underlying biology, but rather due to several more subtle factors. Blacks in WHAS are different from whites along a number of sociological and health measures, and sample size was somewhat limited. Moreover, we did not find that each race was its own best RP, but rather that findings changed unpredictably as the race of the RP changed. Additionally, results were inconsistent across various measures of performance. Accordingly, what we are seeing appears to be noise in the data and fine-scale complexity, and we do not expect our results to be generalizable to the effect of race on RP performance in other populations. For precisely this reason, our race results serve as an important caution in terms of the general applicability of one RP to any other: while most of our results are relatively robust to differences in RP, it is clearly possible to choose RPs that lead to different overall conclusions, and not always easy to predict exactly what these differences will be.

The only clear finding here that would indicate that it is best to use an RP that is different than the study population is that younger and healthier RPs generally perform better. However, it would not appear to be wise to use an RP of young individuals that is too different in other ways (race, sex, country, etc.) from the study population, as indicated by the complex interactions observed. The difference between a young population and the full population is clear but modest, and when a young RP is not available from within the study population we would recommend using the full study population as the RP rather than choosing an external young population.

Interacting with this, there was often a contrast in results for predictions of age versus health outcomes in depending on RP. This difference could reflect the fact that many age-related changes in physiology may be adaptive and protective, given other changes. Older individuals may thus differ in their biomarker profiles from young individuals in some ways that are pathological and other ways that are adaptive. Whether it is best to use a younger reference population may thus depend on a study’s context, particularly on the extent to which it may reflect adaptive versus pathological changes with aging.

The most difficult question likely to arise in practice is what RP to choose for a small study that cannot provide its own. If the study population is broadly representative of the population at large, it might be feasible to choose a subsample from NHANES (which is publicly available) as RP, but this appears not to be advisable if the study population has any particularities, as they might make such an inference problematic. Luckily, the lack of sensitivity to sample size suggests that even many small studies (50+ participants) may be able to provide their own RPs.

While the differences based on RP presented here are mostly minor, the importance of these minor differences depends on context. If we simply wish to show that *D*_*M*_ significantly predicts health outcomes, choice of RP is not important. In contrast, we have observed J-shaped trajectories of *D*_*M*_ with age as opposed to the monotonic increases we would predict [15], and we believe the left tail of the J-shape is due to an imperfect estimation of *μ*, the vector of mean biomarker values. This suggests a more general limit of this study: we are estimating the “optimal” combination of biomarkers based on the mean combination. These two are likely close but not identical, and further work remains to find ways to better estimate optimal *μ* rather than mean *μ*.

## Conclusions

This study provides support for the biological interpretation of *D*_*M*_ as physiological dysregulation (via the better performance of younger, healthier RPs, as predicted) and for the interpretation of physiological dysregulation as an emergent property reflecting the state of complex regulatory networks (via the relative insensitivity of *D*_*M*_ to biomarker choice, and its improving performance with inclusion of more biomarkers). In combination with previous studies, the following key predictions for *D*_*M*_ have now been confirmed: (a) *D*_*M*_ increases with age within individuals [13,14,15]; (b) *D*_*M*_ predicts mortality, frailty, and chronic diseases independently of age [15]; (c) *D*_*M*_ functions similarly in different human populations and even in birds [14,15,30]; (d) *D*_*M*_ is relatively insensitive to which biomarkers are included [13,14,15]; (e) predictive power of *D*_*M*_ improves with the number of biomarkers included [13,14,30]; and (f) predictive power of *D*_*M*_ improves when a younger, healthier RP is used. Given the sum of this evidence, we believe that generalized use of *D*_*M*_ as a measure of physiological dysregulation is now justified across a wide range of contexts, including clinically, in studies of population health, in studies of aging, and as a measure of body condition in an ecological context. The details of the results of this study suggest that in most contexts, *D*_*M*_ can be applied without detailed consideration of biomarker choice or of definition of the RP. However, for small sample sizes or highly specific and particular study populations, we recommend that researchers perform sensitivity analyses to confirm that results do not depend heavily on the choice of RP, and we recommend caution over-interpreting fine-scale changes in *D*_*M*_, particularly in the lower part of its range, until more robust methods of defining an optimal biomarker profile are identified.

## Supporting Information

S1 Text Supporting Materials and Methods. Particularly includes details of measures of health status
(DOCX)

S1 Table Biomarkers used, their mean values by data set, and reference ranges (XLSX)

S1 Fig Mean biomarkers values for BLSA in relation to reported reference ranges. Mean values for each biomarker were normalized according to the reported minimal and maximal normal values, represented by the vertical lines. For biomarker with only one specified normal value, the other vertical line represents minimal or maximal value for the data set (see S1 Table for details).
(TIF)

S2 Fig Mean biomarkers values for WHAS in relation to reported reference ranges. Mean values for each biomarker were normalized according to the reported minimal and maximal normal values, represented by the vertical lines. For biomarker with only one specified normal value, the other vertical line represents minimal or maximal value for the data set (see S1 Table for details).
(TIF)

S3 Fig Mean biomarkers values for InCHIANTI in relation to reported reference ranges. Mean values for each biomarker were normalized according to the reported minimal and maximal normal values, represented by the vertical lines. For biomarker with only one specified normal value, the other vertical line represents minimal or maximal value for the data set (see S1 Table for details).
(TIF)

S4 Fig Correlation between biomarkers in the BLSA data. The magnitude of the correlation between two markers is indicated by the color (scale on the right) and the width of the ellipse shown (a narrow ellipse indicating a stronger correlation), while the tilt shows the sign.
(TIF)

S5 Fig Correlation between biomarkers in the WHAS data. The magnitude of the correlation between two markers is indicated by the color (scale on the right) and the width of the ellipse shown (a narrow ellipse indicating a stronger correlation), while the tilt shows the sign.
(TIF)

S6 Fig Correlation between biomarkers in the InCHIANTI data. The magnitude of the correlation between two markers is indicated by the color (scale on the right) and the width of the ellipse shown (a narrow ellipse indicating a stronger correlation), while the tilt shows the sign.
(TIF)

S7 Fig Correlation between biomarkers in the NHANES data. The magnitude of the correlation between two markers is indicated by the color (scale on the right) and the width of the ellipse shown (a narrow ellipse indicating a stronger correlation), while the tilt shows the sign.
(TIF)

S8 Fig Contribution of individual biomarkers to pairwise *D*_*M*_ correlation for the BLSA dataset. The X-axis represents the number of biomarkers per group (*N*_*bm*_) and the Y-axis reports the coefficient (*β*) from a linear regression of the *D*_*M*_ pairwise correlations on *N*_*bm*_. *βs* represent the deviation from the average correlation when a given biomarker is included in the calculation of *D*_*M*_; positive values thus indicate improved performance of *D*_*M*_, and negative values decreased performance. Colors indicate the magnitude of *p*-values, with darker red being more significant and white not significant.
(TIF)

S9 Fig Contribution of individual biomarkers to pairwise *D*_*M*_ correlation for the WHAS dataset. The X-axis represents the number of biomarkers per group (*N*_*bm*_) and the Y-axis reports the coefficient (*β*) from a linear regression of the *D*_*M*_ pairwise correlations on *N*_*bm*_. *βs* represent the deviation from the average correlation when a given biomarker is included in the calculation of *D*_*M*_; positive values thus indicate improved performance of *D*_*M*_, and negative values decreased performance. Colors indicate the magnitude of *p*-values, with darker red being more significant and white not significant. Empty panels are shown for biomarkers with no data for this particular data set (see text and Table 1 for details).
(TIF)

S10 Fig Contribution of individual biomarkers to pairwise *D*_*M*_ correlation for the InCHIANTI dataset. The X-axis represents the number of biomarkers per group (*N*_*bm*_) and the Y-axis reports the coefficient (*β*) from a linear regression of the *D*_*M*_ pairwise correlations on *N*_*bm*_. *βs* represent the deviation from the average correlation when a given biomarker is included in the calculation of *D*_*M*_; positive values thus indicate improved performance of *D*_*M*_, and negative values decreased performance. Colors indicate the magnitude of *p*-values, with darker red being more significant and white not significant.
(TIF)

S11 Fig Contribution of individual biomarkers to pairwise *D*_*M*_ correlation for the NHANES dataset. The X-axis represents the number of biomarkers per group (*N*_*bm*_) and the Y-axis reports the coefficient (*β*) from a linear regression of the *D*_*M*_ pairwise correlations on *N*_*bm*_. *βs* represent the deviation from the average correlation when a given biomarker is included in the calculation of *D*_*M*_; positive values thus indicate improved performance of *D*_*M*_, and negative values decreased performance. Colors indicate the magnitude of *p*-values, with darker red being more significant and white not significant. Empty panels are shown for biomarkers with no data for this particular data set (see text and Table 1 for details).
(TIF)

S12 Fig Contribution of individual biomarkers to change in variance in age explained by *D*_*M*_, for the BLSA dataset. The X-axis represents the number of biomarkers per group (*N*_*bm*_), while the Y-axis reports the change in how much variance in age is predicted by *D*_*M*_ with the inclusion of the given biomarker, based on a meta-regression of all the R-squareds calculated for individual quadratic regressions of age and *D*_*M*_. Colors indicate the magnitude of *p*-values, with darker red being more significant and white not significant.
(TIF)

S13 Fig Contribution of individual biomarkers to change in variance in age explained by *D*_*M*_, for the WHAS dataset. The X-axis represents the number of biomarkers per group (*N*_*bm*_), while the Y-axis reports the change in how much variance in age is predicted by *D*_*M*_ with the inclusion of the given biomarker, based on a meta-regression of all the R-squareds calculated for individual quadratic regressions of age and *D*_*M*_. Colors indicate the magnitude of *p*-values, with darker red being more significant and white not significant. Empty panels are shown for biomarkers with no data for this particular data set (see text and Table 1 for details).
(TIF)

S14 Fig Contribution of individual biomarkers to change in variance in age explained by *D*_*M*_, for the InCHIANTI dataset. The X-axis represents the number of biomarkers per group (*N*_*bm*_), while the Y-axis reports the change in how much variance in age is predicted by *D*_*M*_ with the inclusion of the given biomarker, based on a meta-regression of all the R-squareds calculated for individual quadratic regressions of age and *D*_*M*_. Colors indicate the magnitude of *p*-values, with darker red being more significant and white not significant.
(TIF)

S15 Fig Contribution of individual biomarkers to change in variance in age explained by *D*_*M*_, for the NHANES dataset. The X-axis represents the number of biomarkers per group (*N*_*bm*_), while the Y-axis reports the change in how much variance in age is predicted by *D*_*M*_ with the inclusion of the given biomarker, based on a meta-regression of all the R-squareds calculated for individual quadratic regressions of age and *D*_*M*_. Colors indicate the magnitude of *p*-values, with darker red being more significant and white not significant. Empty panels are shown for biomarkers with no data for this particular data set (see text and Table 1 for details).
(TIF)

S16 Fig Effects of RPs age intervals on prediction of age and health outcomes. The study population represented here is the full NHANES data set stratified by sex as indicated, with RPs of differing age intervals (also NHANES). The width of the rectangle represents the average effect size (here the correlation between *D*_*M*_ and age) among significant analyses, relative to the effect size of the rectangle in the leftmost column (entire study population as its own RP). The percentage of significant p-values is represented by the height of shading within the rectangle, the shading colour represents the direction of the effect (blue is a positive effect), and the hue represents the average p-value among the significant *p*-values, with darker hues indicating lower *p*-values.
(TIF)

S17 Fig Effects of RPs drawn from external young populations on prediction of age and health outcomes. The study population represented here is the full InCHIANTI data set with RPs of young individuals from each of the three data sets. The width of the rectangle represents the average effect size among significant analyses, relative to the effect size of the rectangle in the leftmost column (entire study population as its own RP). The percentage of significant p-values is represented by the height of shading within the rectangle, the shading colour represents the direction of the effect (blue is a positive effect), and the hue represents the average p-value among the significant *p*-values, with darker hues indicating lower *p*-values.
(TIF)

S18 Fig Effects of RP race composition on prediction of age and health outcomes. The study population represented here is the full WHAS data set using mixed, white-only, and black-only RPs (also WHAS). The width of the rectangle represents the average effect size among significant analyses, relative to the effect size of the rectangle in the leftmost column (entire study population as its own RP). The percentage of significant p-values is represented by the height of shading within the rectangle, the shading colour represents the direction of the effect (blue is a positive effect), and the hue represents the average p-value among the significant *p*-values, with darker hues indicating lower *p*-values.
(TIF)

S19 Fig Effects of RP sex composition on prediction of age and health outcomes. The study population represented here is the full InCHIANTI data set, varying the sex of the RP (also InCHIANTI). The width of the rectangle represents the average effect size among significant analyses, relative to the effect size of the rectangle in the leftmost column (entire study population as its own RP). The percentage of significant p-values is represented by the height of shading within the rectangle, the shading colour represents the direction of the effect (blue is a positive effect), and the hue represents the average p-value among the significant *p*-values, with darker hues indicating lower *p*-values.
(TIF)
