# Supplementary material for: Statistical Distance as a Measure of Physiological Dysregulation Is Largely Robust to Variation in Its Biomarker Composition
Source: PLoS One. 2015 Apr 13;10(4):e0122541. doi: 10.1371/journal.pone.0122541 (PMC4395377; doi:10.1371/journal.pone.0122541)
Supplement: S1 Text — Particularly includes details of measures of health status (DOCX) [file pone.0122541.s001.docx]

Supporting Information for

**Statistical distance as a measure of physiological dysregulation is largely robust to variation in its biomarker composition**

Alan A. Cohen**,** Qing Li, Emmanuel Milot, Maxime Leroux, Samuel Faucher, Vincent Morissette-Thomas, Véronique Legault, Linda P. Fried, and Luigi Ferrucci

**SUPPORTING MATERIALS AND METHODS**

**HEALTH STATUS MEASURES**

Health status measures were not available for BLSA. Type and availability of data varied substantially between WHAS and InCHIANTI; variables were defined as follows:

*CARDIOVASCULAR DISEASE*

For InCHIANTI, cardiovascular disease (CVD) was considered positive when at least one of the following was present: angina pectoris, myocardial infarction, congestive heart failure (CHF), stroke, peripheral arterial disease (PAD) or severe stenosis on coronary angiography. The presence of angina pectoris was defined as definite (score of 1) when there was use of organic nitrates and either one of the following: self-reported diagnosis, documentation or Rose Angina Questionnaire (ROSANG) score. If only one of medication, self-report, documentation or ROSANG score was present, a possible diagnosis was given (score of 0.5). We only retained angina pectoris diagnosed within the previous year or under current treatment. The presence of myocardial infarction was considered definite when there were signs of necrosis on electrocardiography (ECG), documented and self-reported diagnosis, aorto-coronary bypass or angioplasty. A possible score was attributed when only self-report was present. Only diagnoses within the previous year were retained. A definite score for CHF was attributed when self-report and physical exam or documentation were present or when any of those three, in addition to medication (diuretics or aldosterone antagonists and angiotensine II antagonists, angiotensin-converting-enzyme inhibitors or digitalis glycosides) or evidence in the ECG exam (presence of necrosis, atrial fibrillation in the cardiac rhythm, incomplete or complete L bundle branch blk in the intraventricular conduction, or L ventricular hypertrophy or overload), was present. A possible CHF score was given when only one of the aforementioned criteria was met. Stroke diagnosis was based on self-report, physical exam and documentation; when two or more criteria were present, a definite score was given while a possible score was attributed when only one diagnostic criterion was met. Transient ischemic attack (self-report) was considered as possible stroke. A definite score for PAD was given to ankle-brachial index (lower blood pressure of the two legs) lower than 0.9. A possible score was attributed to evidence in the physical exam, documentation or Rose PAD questionnaire. The Rose PAD questionnaire score was calculated according to responses to Rose claudication items (pain in legs while walking, standing still or sitting, pain in the calf and pain in the leg or foot at night). Diagnoses for angina pectoris, CHF, stroke, PAD and severe stenosis were adjusted across follow-up visits, as described above. In WHAS, CVD was considered positive when at least one the following was present: myocardial infarction, angina pectoris, CHF, heart disease, or stroke. All diagnoses were based on self-report. Thus, in InCHIANTI each individual had a score or 0, 0.5, or 1 at each time point; in WHAS, each individual had a score of 0 or 1 at baseline.

*FRAILTY*

We used Fried’s frailty criteria1 to assign a number of criteria (between 0 and 5). This measure was available at baseline in InCHIANTI and at each visit in WHAS. Fried’s frailty criteria are unintentional weight loss, fatigue, reduced grip strength, reduced physical activity, and low gait speed; fulfilling three or more of these criteria indicates clinical frailty, and fulfilling 1-2 indicates a pre-frail state. We use the number of criteria rather than frailty state in order to maximize statistical power; similar results are obtained using a dichotomous frail-non-frail outcome.

*COMORBIDITIES*

For InCHIANTI, the number of comorbidities included definite diagnoses for the following: cancer, chronic liver disease (self-report), angina pectoris, myocardial infarction, CHF, stroke, diabetes mellitus (defined as blood glucose ≥140 mg/dL or use of diabetes drugs), chronic bronchitis or emphysema, profoundly impaired renal function (creatinine clearance assessed via Cockcroft-Gault formula ≤30 mL/min), severe stenosis, kidney failure (self-report), deep venous thrombosis (DVT) and thyroid disease. For WHAS, the number of comorbidities included any positive diagnosis for cancer, angina pectoris, myocardial infarction, CHF, stroke, diabetes mellitus, heart disease, and lung disease, all of which were based on self-reported diagnosis. Thus, for InCHIANTI each individual had a score between 0 and 13 at each time point; for WHAS, each individual had a score between 0 and 9 at baseline.

**SUPORTING REFERENCES**

1. Medical Council of Canada (2010) Objectives for the Qualifying Examination, 3rd Edition.

2. Kratz A, Ferraro M, Sluss PM, Lewandrowski KB (2004) Case records of the Massachusetts General Hospital. Weekly clinicopathological exercises. Laboratory reference values. N Engl J Med 351: 1548-1563.

3. Fernandez-Real JM, Vayreda M, Richart C, Gutierrez C, Broch M, et al. (2001) Circulating interleukin 6 levels, blood pressure, and insulin sensitivity in apparently healthy men and women. J Clin Endocrinol Metab 86: 1154-1159.

4. Iron Disorders Institute’s Scientific & Medical Advisory Board (2010).

5. Canadian Health Measures Survey Physician Advisory Committee (2010).

6. Dicken W, Scott F (2002) Blood Chemistry and CBC Analysis: Clinical Laboratory Testing from a Functional Perspective.: Bear Mountain.

7. Pagana K, Pagana T (2010) Mosby’s Manual of Diagnostic and Laboratory Tests, 4th ed. St. Louis: Mosby Elsevier.
