# Supplementary figures and images for: Statistical Distance as a Measure of Physiological Dysregulation Is Largely Robust to Variation in Its Biomarker Composition
Source: PLoS One. 2015 Apr 13;10(4):e0122541. doi: 10.1371/journal.pone.0122541 (PMC4395377; doi:10.1371/journal.pone.0122541)

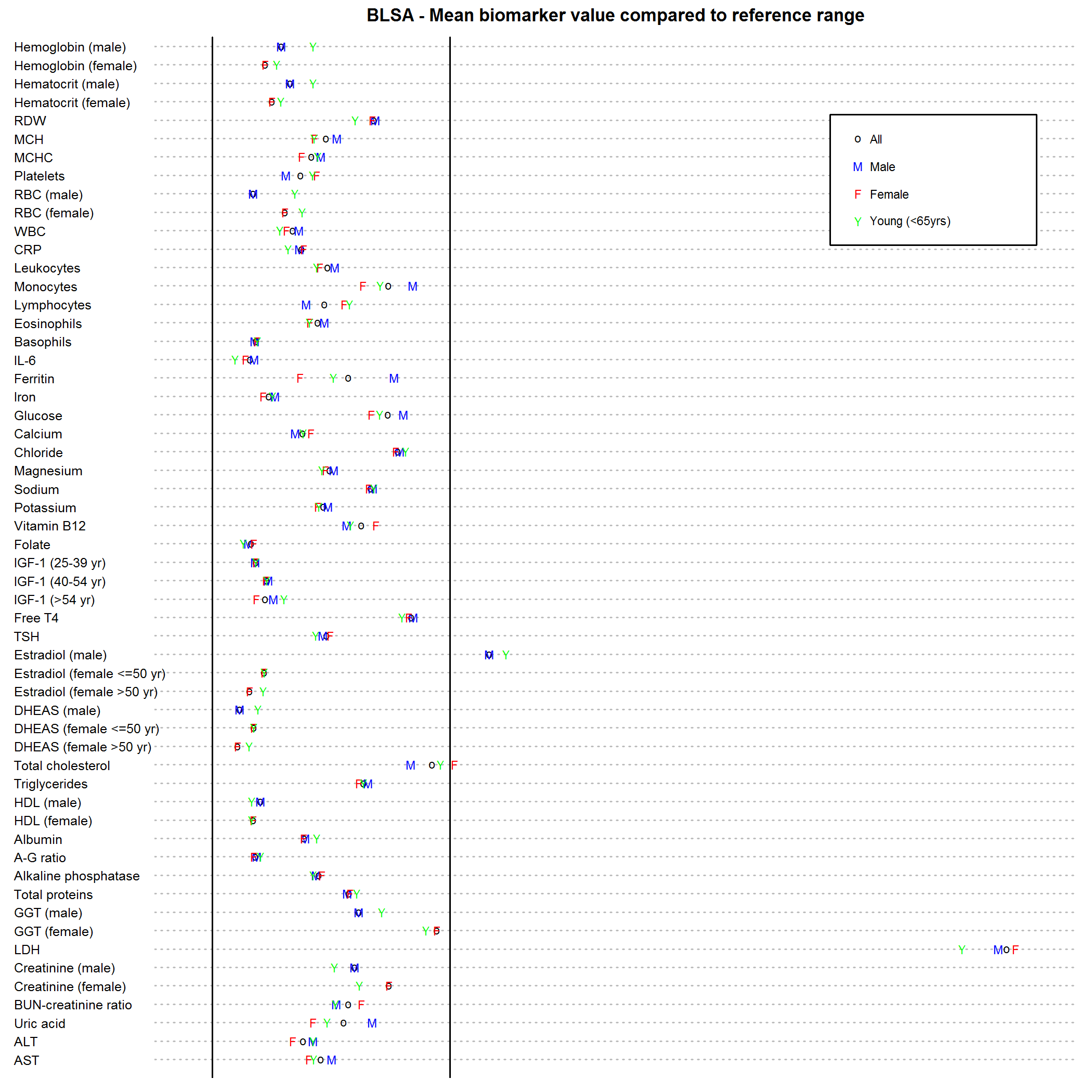

Supplement: S1 Fig — Mean values for each biomarker were normalized according to the reported minimal and maximal normal values, represented by the vertical lines. For biomarker with only one specified normal value, the other vertical line represents minimal or maximal value for the data set (see S1 Table for details). (TIF) [file pone.0122541.s003.tif]

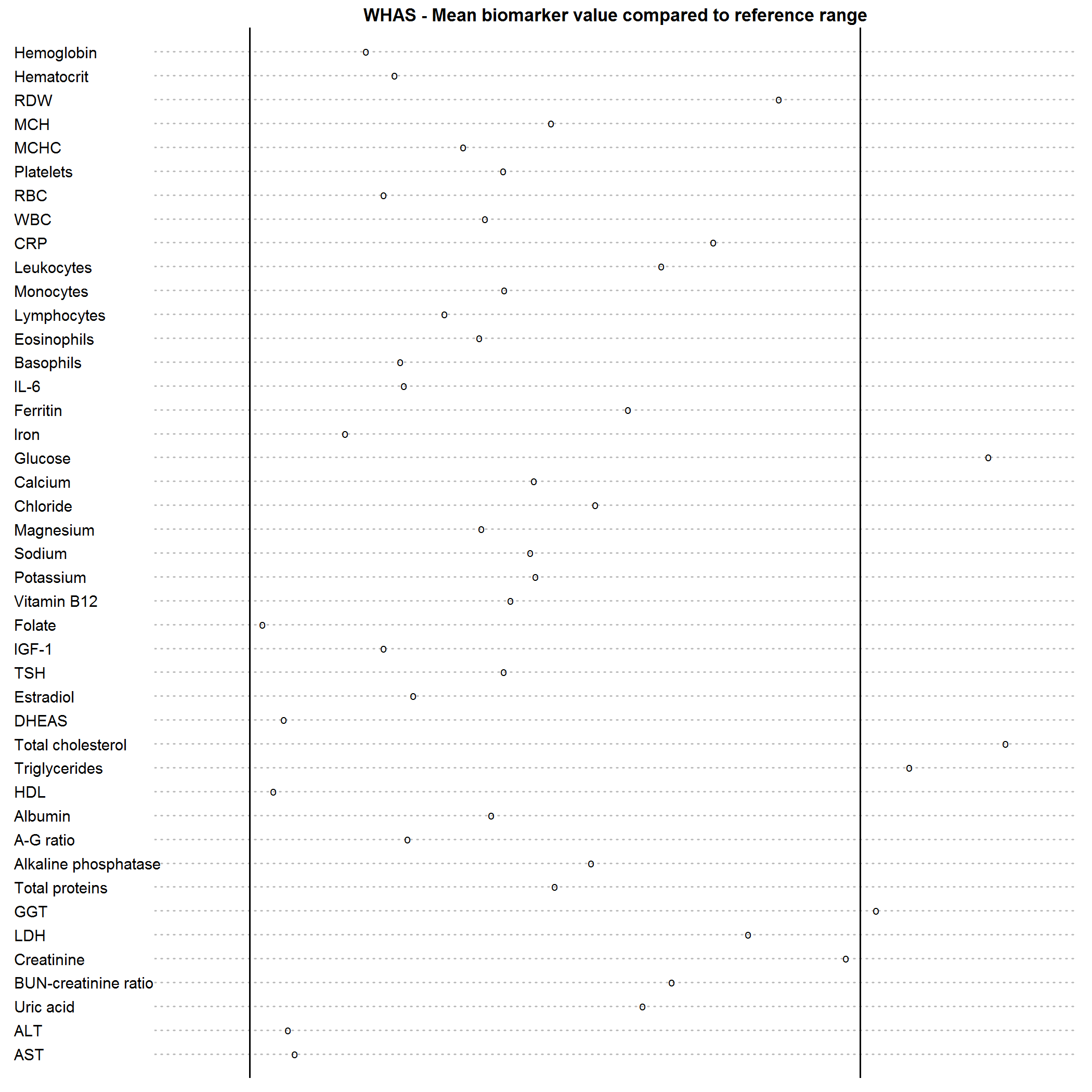

Supplement: S2 Fig — Mean values for each biomarker were normalized according to the reported minimal and maximal normal values, represented by the vertical lines. For biomarker with only one specified normal value, the other vertical line represents minimal or maximal value for the data set (see S1 Table for details). (TIF) [file pone.0122541.s004.tif]

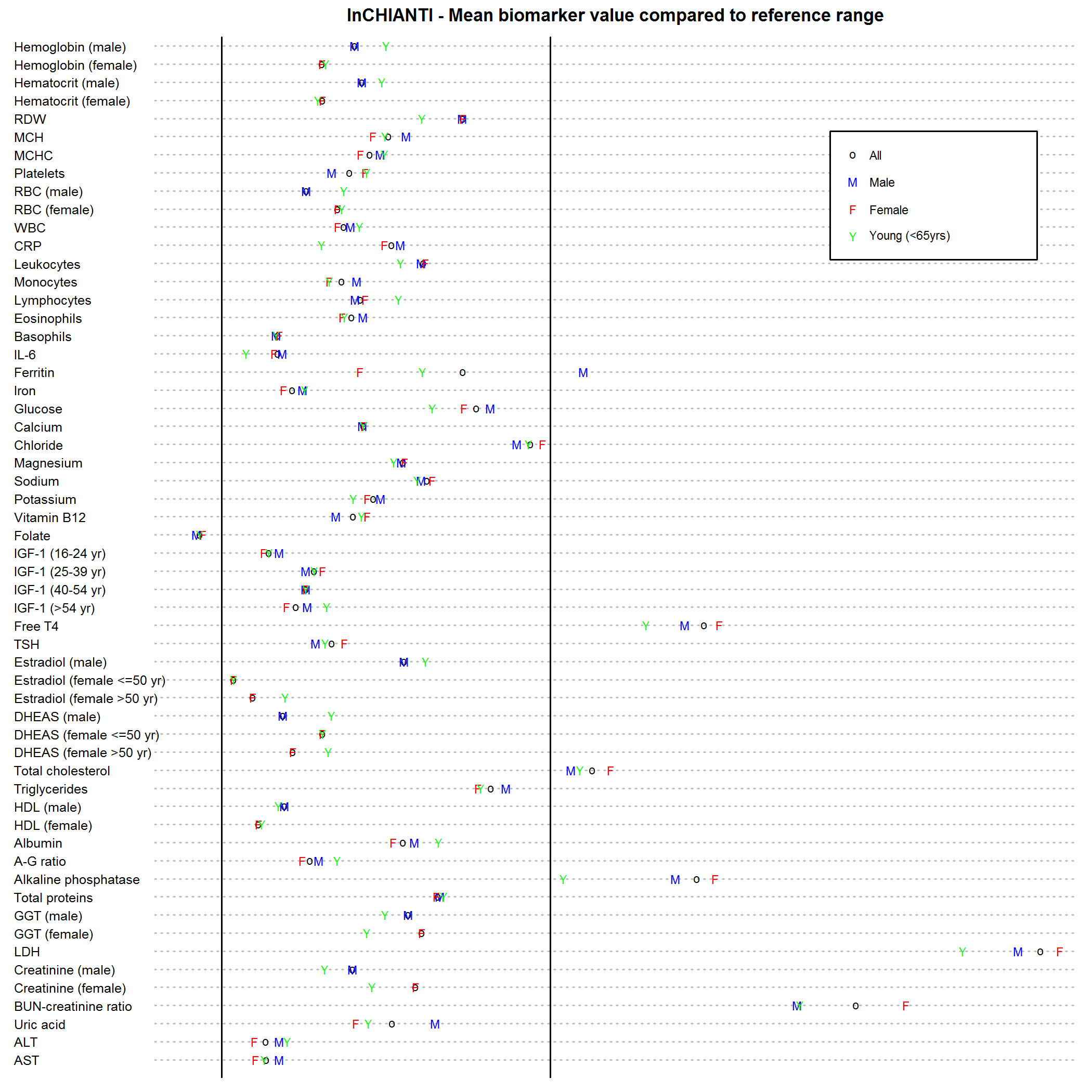

Supplement: S3 Fig — Mean values for each biomarker were normalized according to the reported minimal and maximal normal values, represented by the vertical lines. For biomarker with only one specified normal value, the other vertical line represents minimal or maximal value for the data set (see S1 Table for details). (TIF) [file pone.0122541.s005.tif]

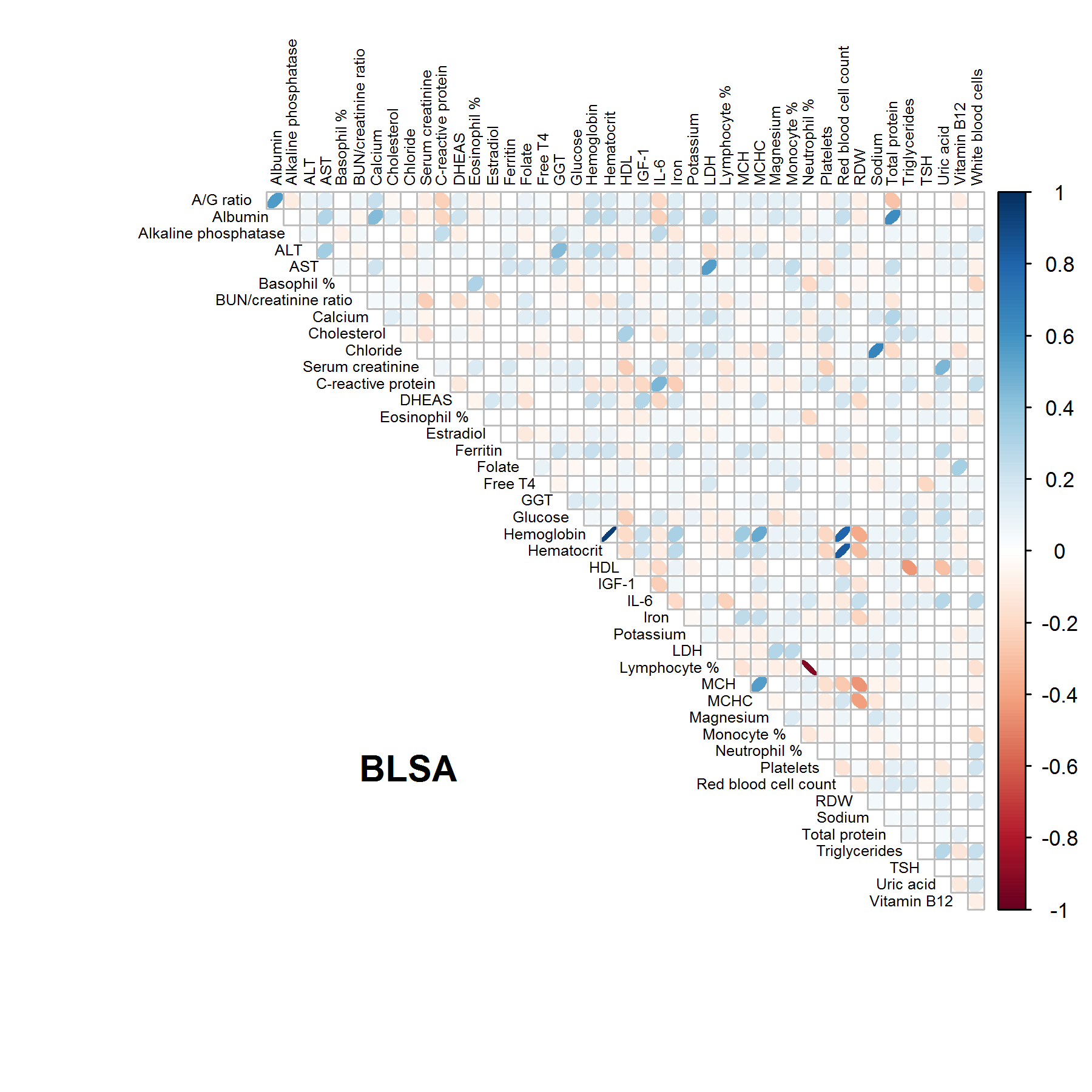

Supplement: S4 Fig — The magnitude of the correlation between two markers is indicated by the color (scale on the right) and the width of the ellipse shown (a narrow ellipse indicating a stronger correlation), while the tilt shows the sign. (TIF) [file pone.0122541.s006.tif]

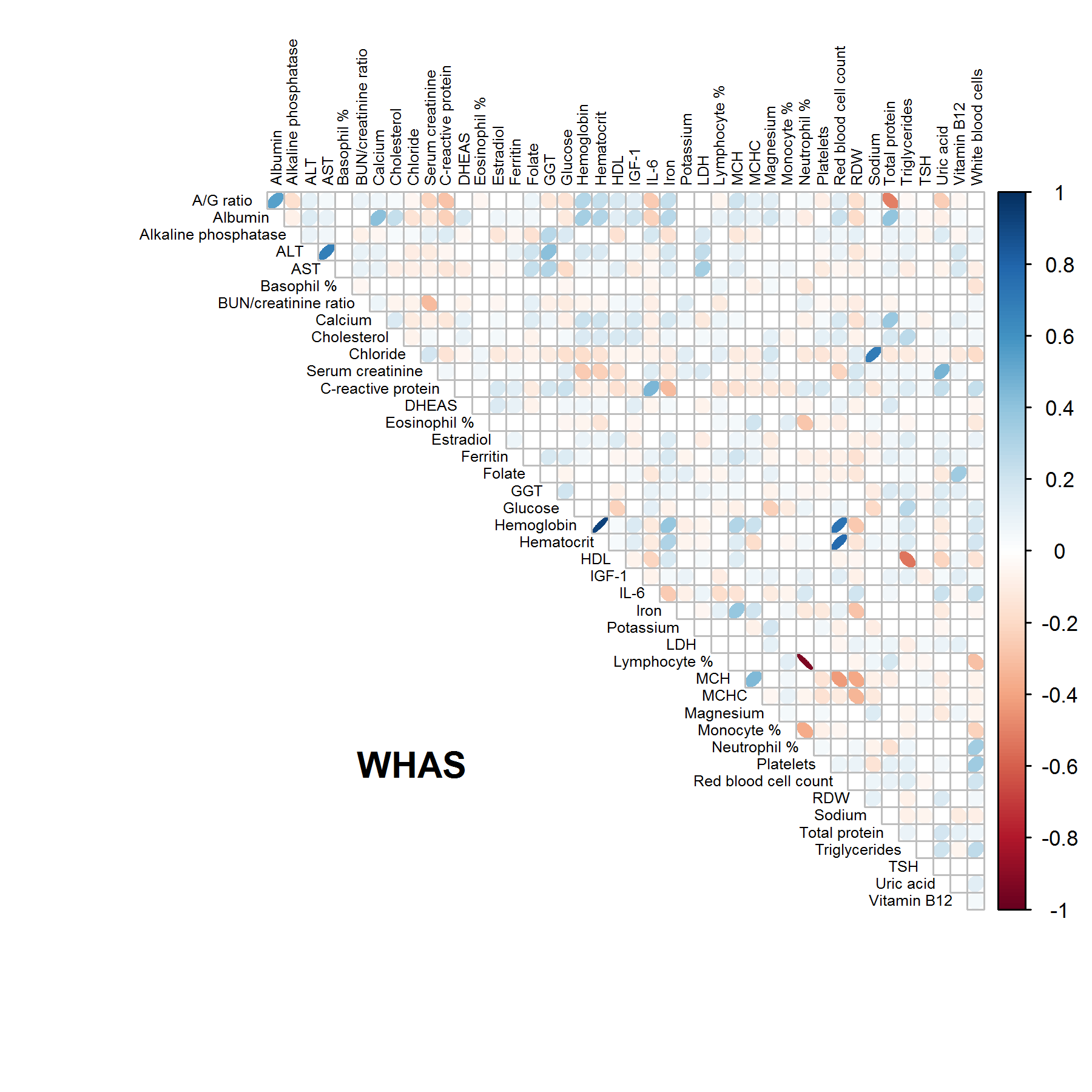

Supplement: S5 Fig — The magnitude of the correlation between two markers is indicated by the color (scale on the right) and the width of the ellipse shown (a narrow ellipse indicating a stronger correlation), while the tilt shows the sign. (TIF) [file pone.0122541.s007.tif]

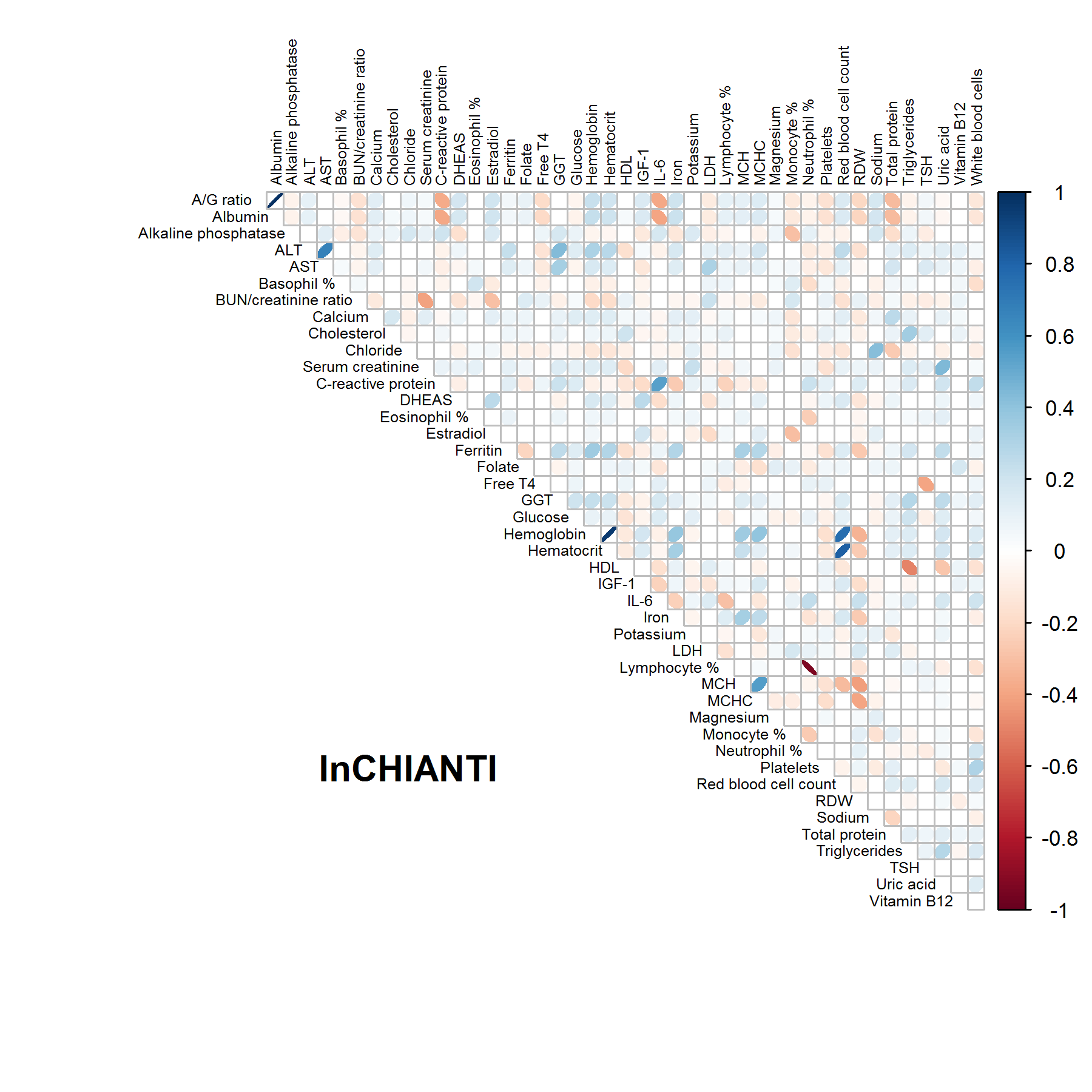

Supplement: S6 Fig — The magnitude of the correlation between two markers is indicated by the color (scale on the right) and the width of the ellipse shown (a narrow ellipse indicating a stronger correlation), while the tilt shows the sign. (TIF) [file pone.0122541.s008.tif]

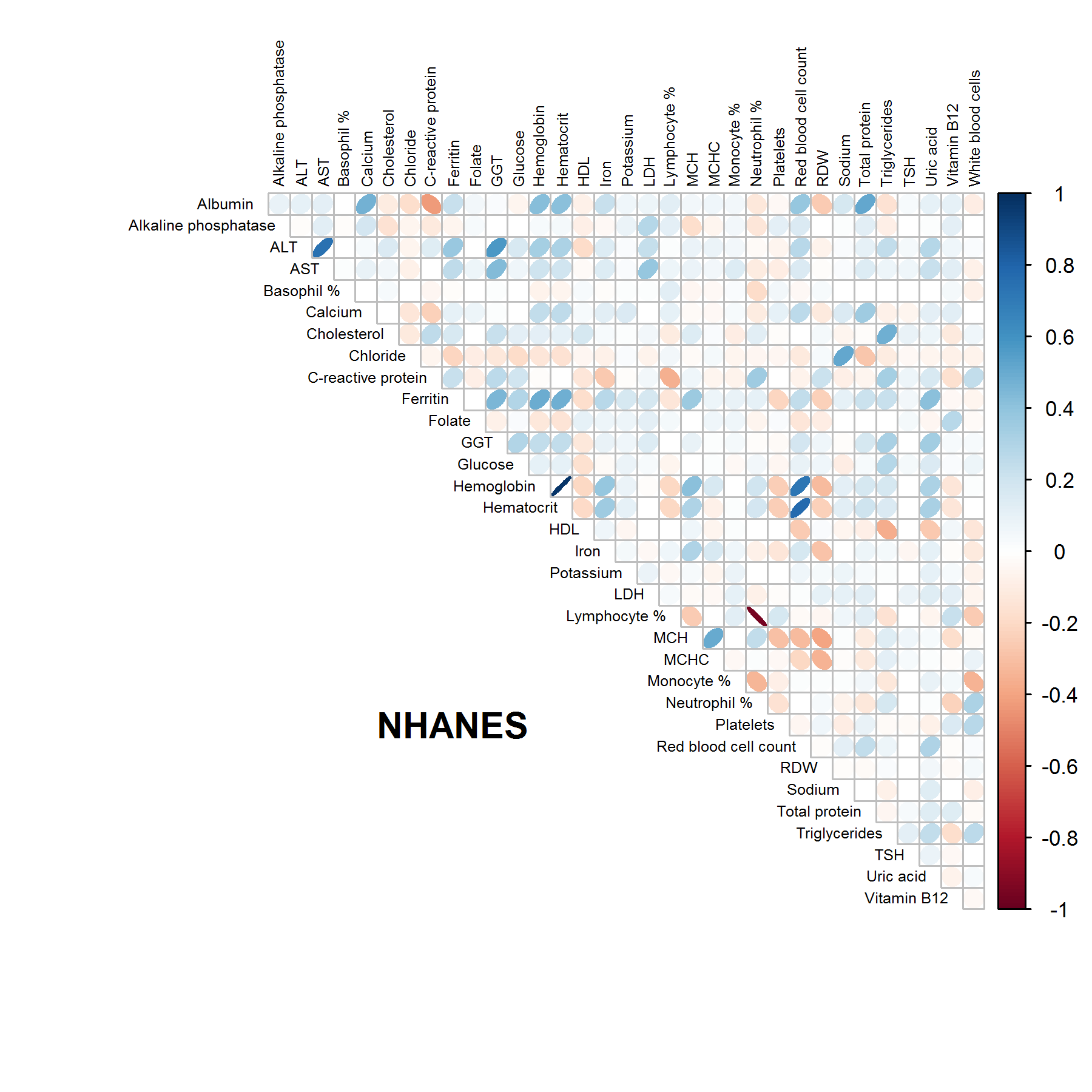

Supplement: S7 Fig — The magnitude of the correlation between two markers is indicated by the color (scale on the right) and the width of the ellipse shown (a narrow ellipse indicating a stronger correlation), while the tilt shows the sign. (TIF) [file pone.0122541.s009.tif]

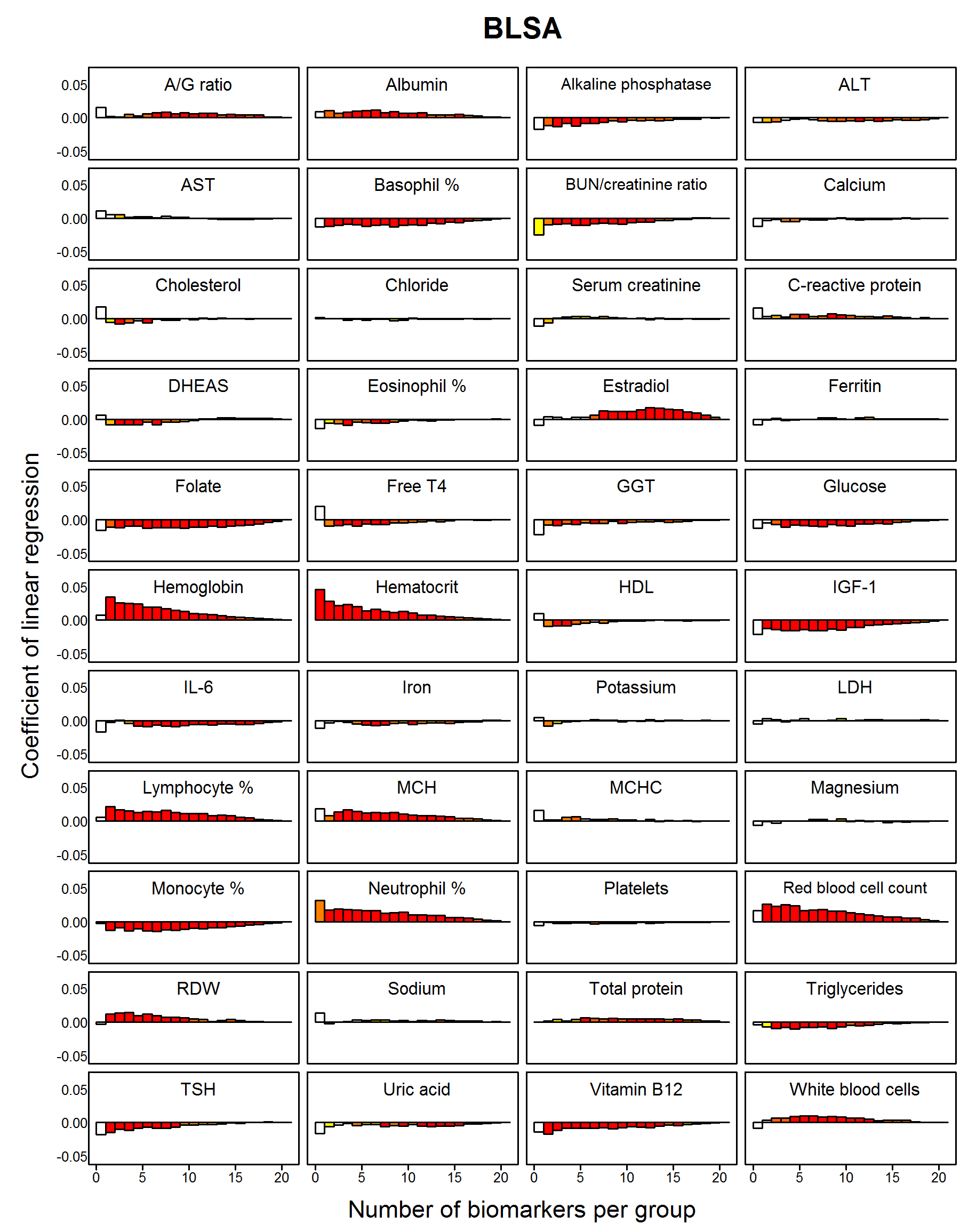

Supplement: S8 Fig — The X-axis represents the number of biomarkers per group (N bm) and the Y-axis reports the coefficient (β) from a linear regression of the D M pairwise correlations on N bm. βs represent the deviation from the average correlation when a given biomarker is included in the calculation of D M; positive values thus indicate improved performance of D M, and negative values decreased performance. Colors indicate the magnitude of p-values, with darker red being more significant and white not significant. (TIF) [file pone.0122541.s010.tif]

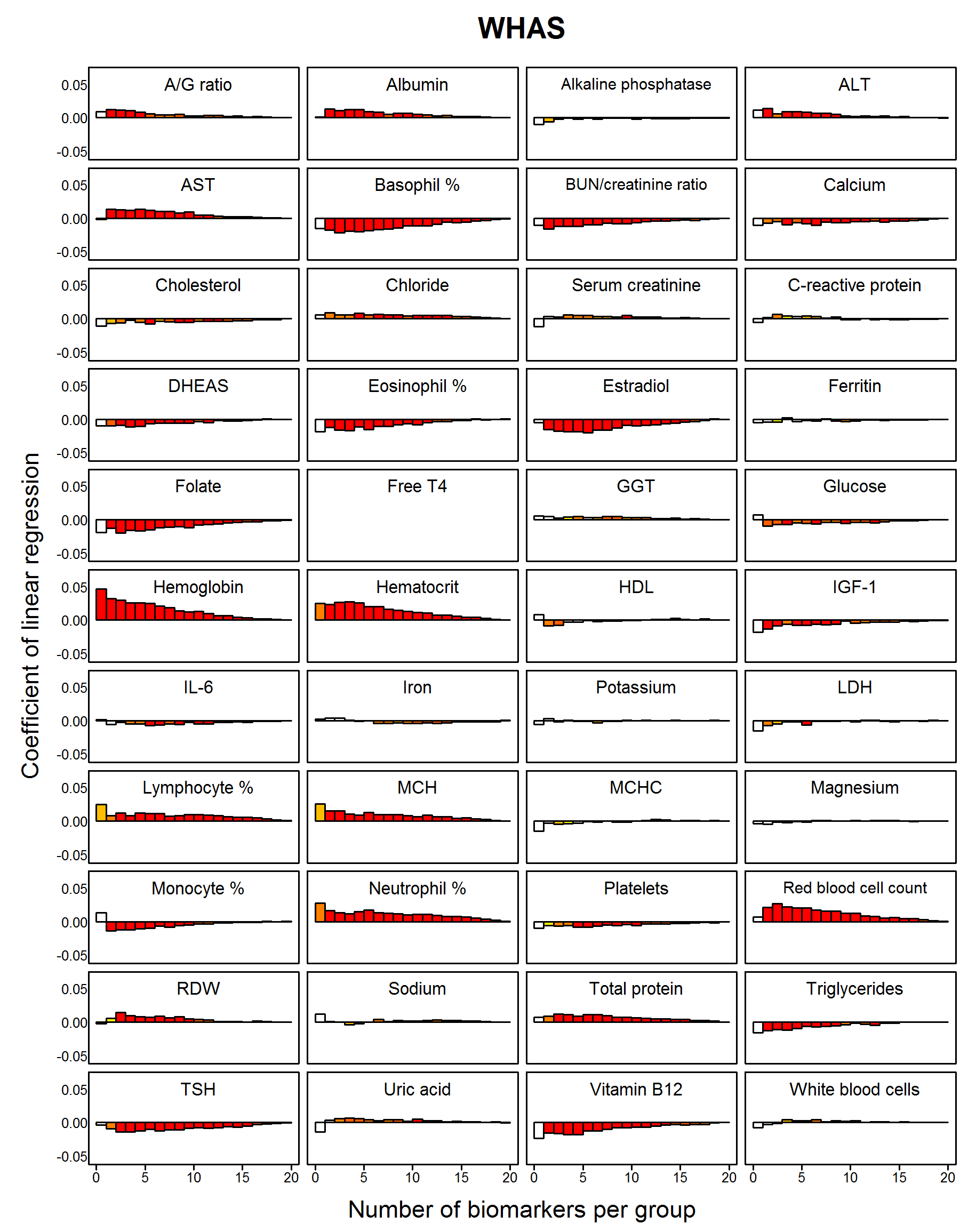

Supplement: S9 Fig — The X-axis represents the number of biomarkers per group (N bm) and the Y-axis reports the coefficient (β) from a linear regression of the D M pairwise correlations on N bm. βs represent the deviation from the average correlation when a given biomarker is included in the calculation of D M; positive values thus indicate improved performance of D M, and negative values decreased performance. Colors indicate the magnitude of p-values, with darker red being more significant and white not significant. Empty panels are shown for biomarkers with no data for this particular data set (see text and Table 1 for details). (TIF) [file pone.0122541.s011.tif]

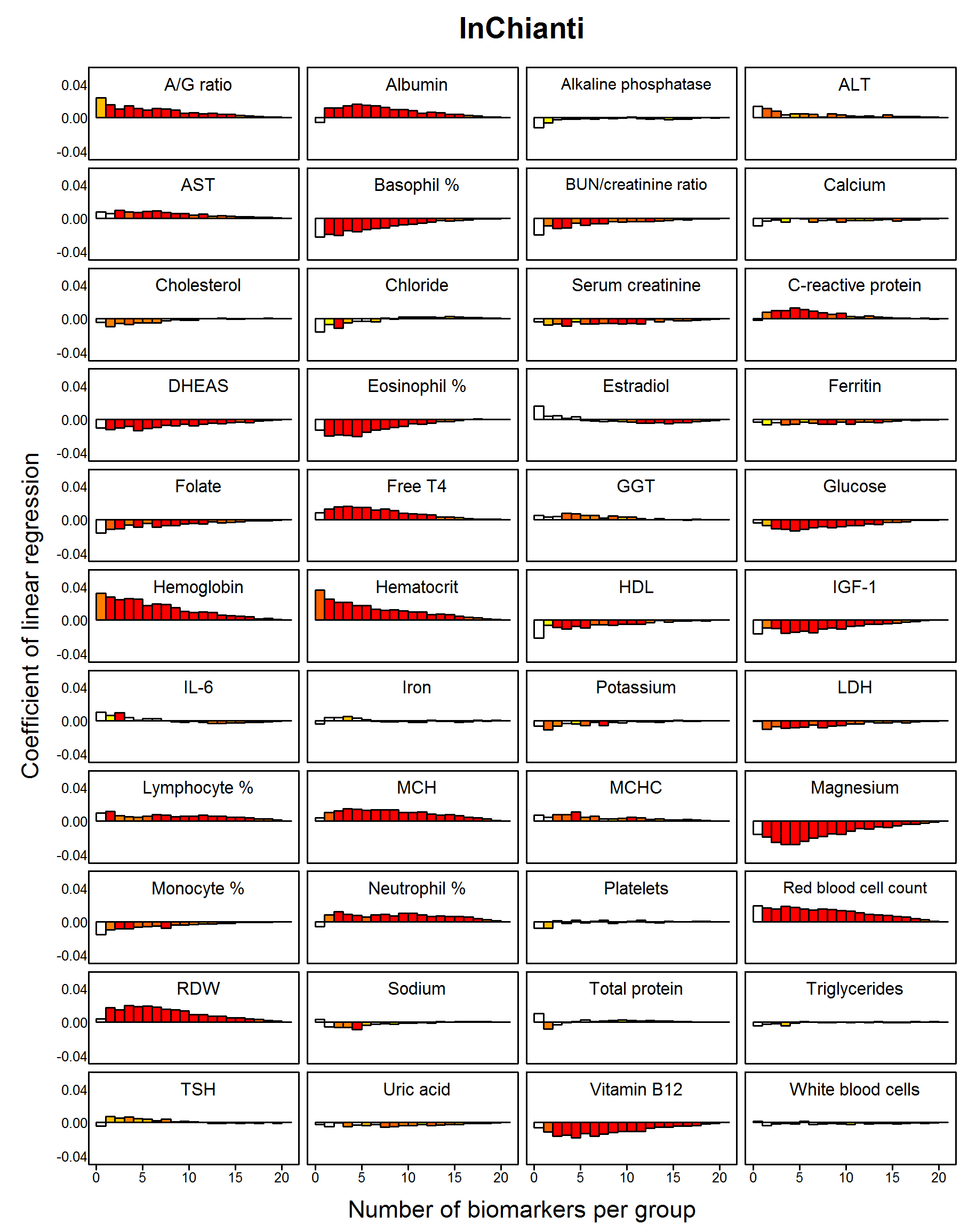

Supplement: S10 Fig — The X-axis represents the number of biomarkers per group (N bm) and the Y-axis reports the coefficient (β) from a linear regression of the D M pairwise correlations on N bm. βs represent the deviation from the average correlation when a given biomarker is included in the calculation of D M; positive values thus indicate improved performance of D M, and negative values decreased performance. Colors indicate the magnitude of p-values, with darker red being more significant and white not significant. (TIF) [file pone.0122541.s012.tif]

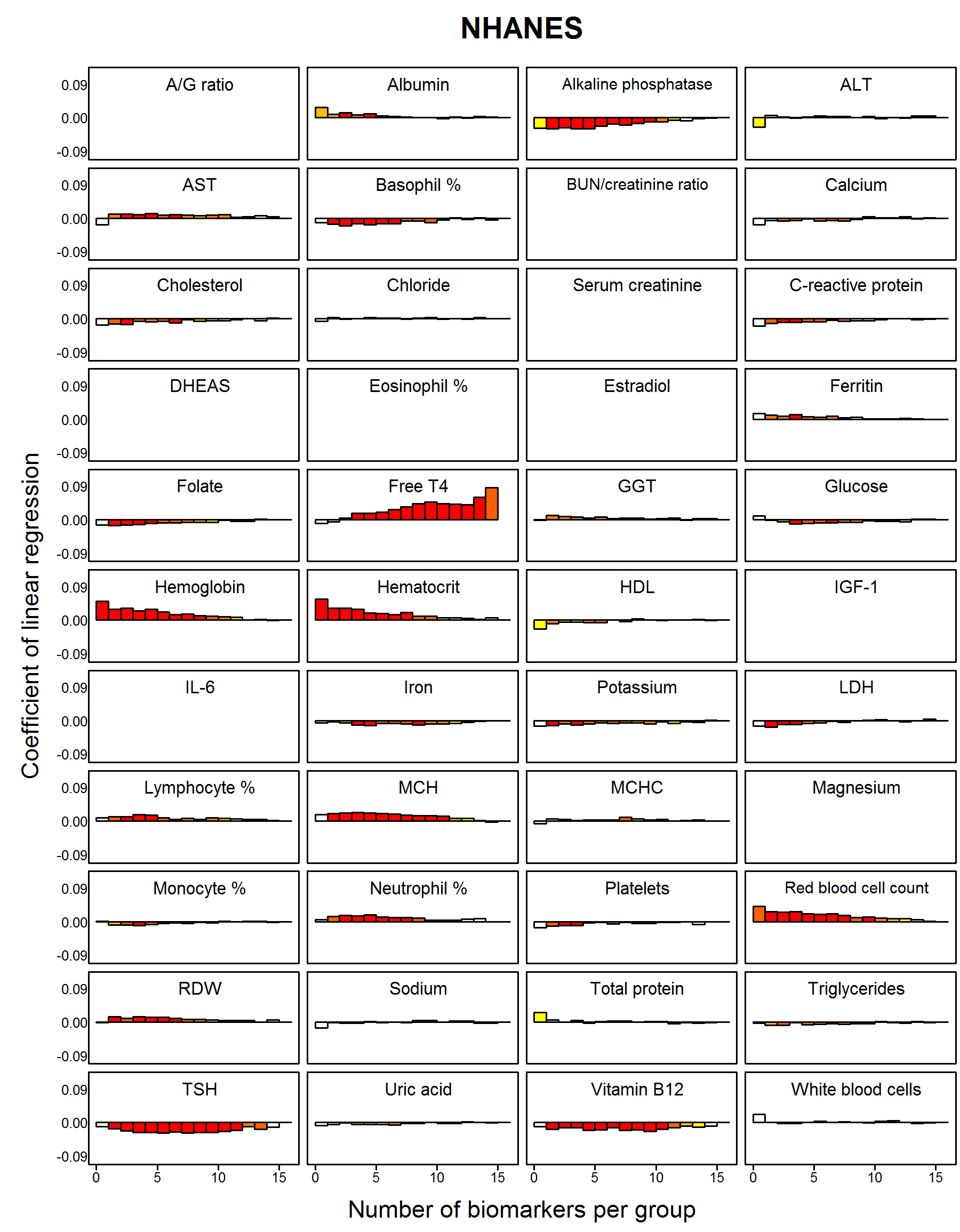

Supplement: S11 Fig — The X-axis represents the number of biomarkers per group (N bm) and the Y-axis reports the coefficient (β) from a linear regression of the D M pairwise correlations on N bm. βs represent the deviation from the average correlation when a given biomarker is included in the calculation of D M; positive values thus indicate improved performance of D M, and negative values decreased performance. Colors indicate the magnitude of p-values, with darker red being more significant and white not significant. Empty panels are shown for biomarkers with no data for this particular data set (see text and Table 1 for details). (TIF) [file pone.0122541.s013.tif]

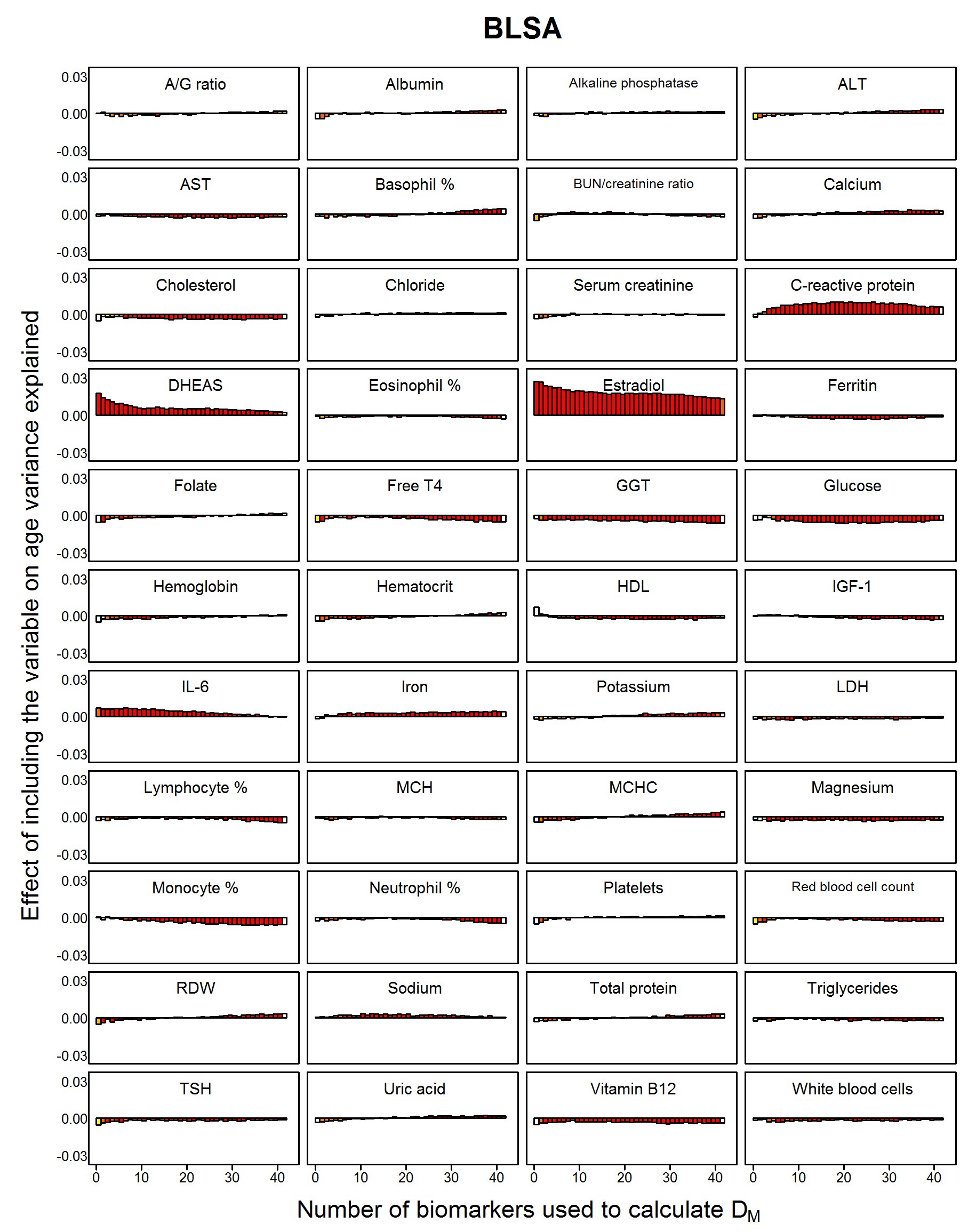

Supplement: S12 Fig — The X-axis represents the number of biomarkers per group (N bm), while the Y-axis reports the change in how much variance in age is predicted by D M with the inclusion of the given biomarker, based on a meta-regression of all the R-squareds calculated for individual quadratic regressions of age and D M. Colors indicate the magnitude of p-values, with darker red being more significant and white not significant. (TIF) [file pone.0122541.s014.tif]

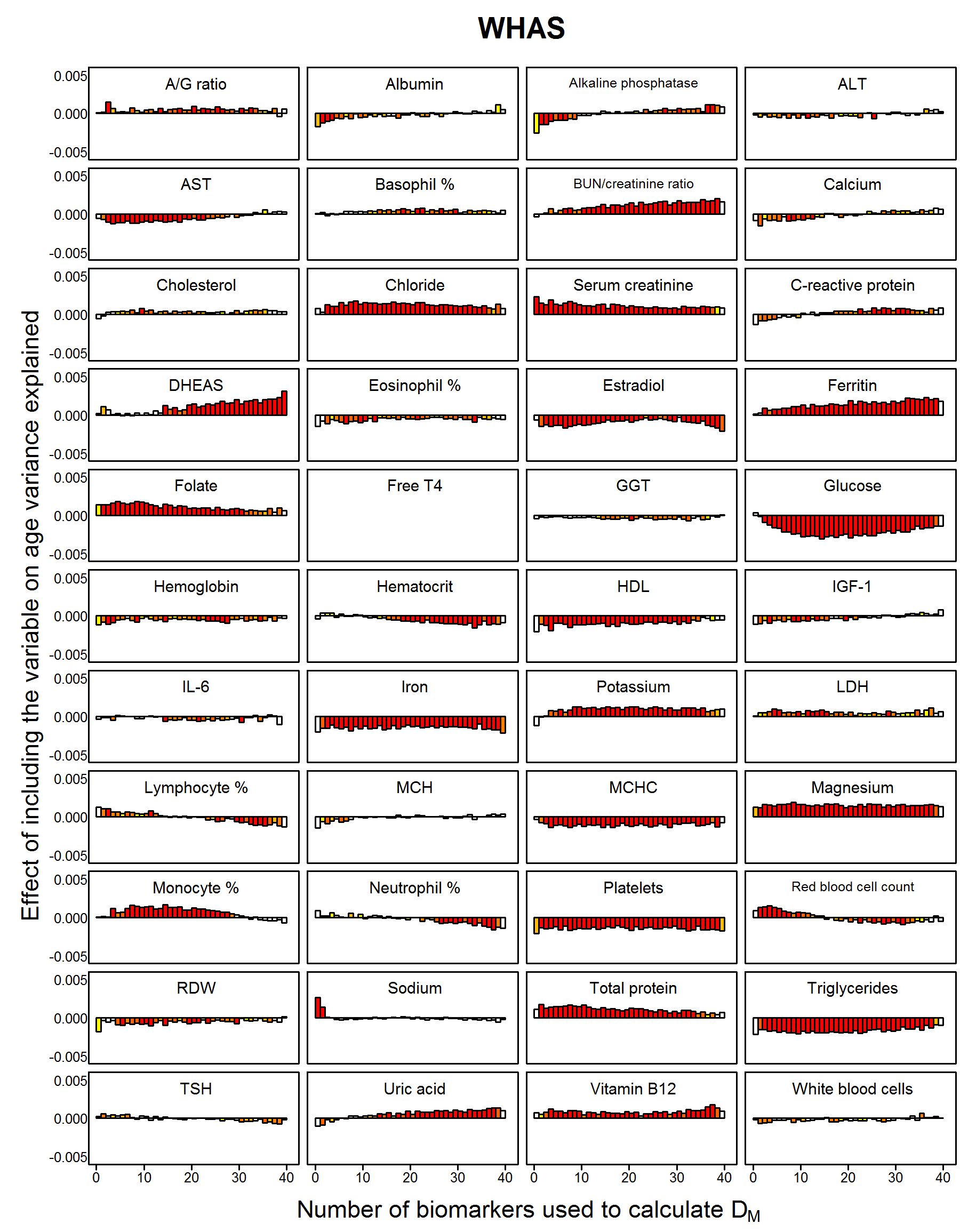

Supplement: S13 Fig — The X-axis represents the number of biomarkers per group (N bm), while the Y-axis reports the change in how much variance in age is predicted by D M with the inclusion of the given biomarker, based on a meta-regression of all the R-squareds calculated for individual quadratic regressions of age and D M. Colors indicate the magnitude of p-values, with darker red being more significant and white not significant. Empty panels are shown for biomarkers with no data for this particular data set (see text and Table 1 for details). (TIF) [file pone.0122541.s015.tif]

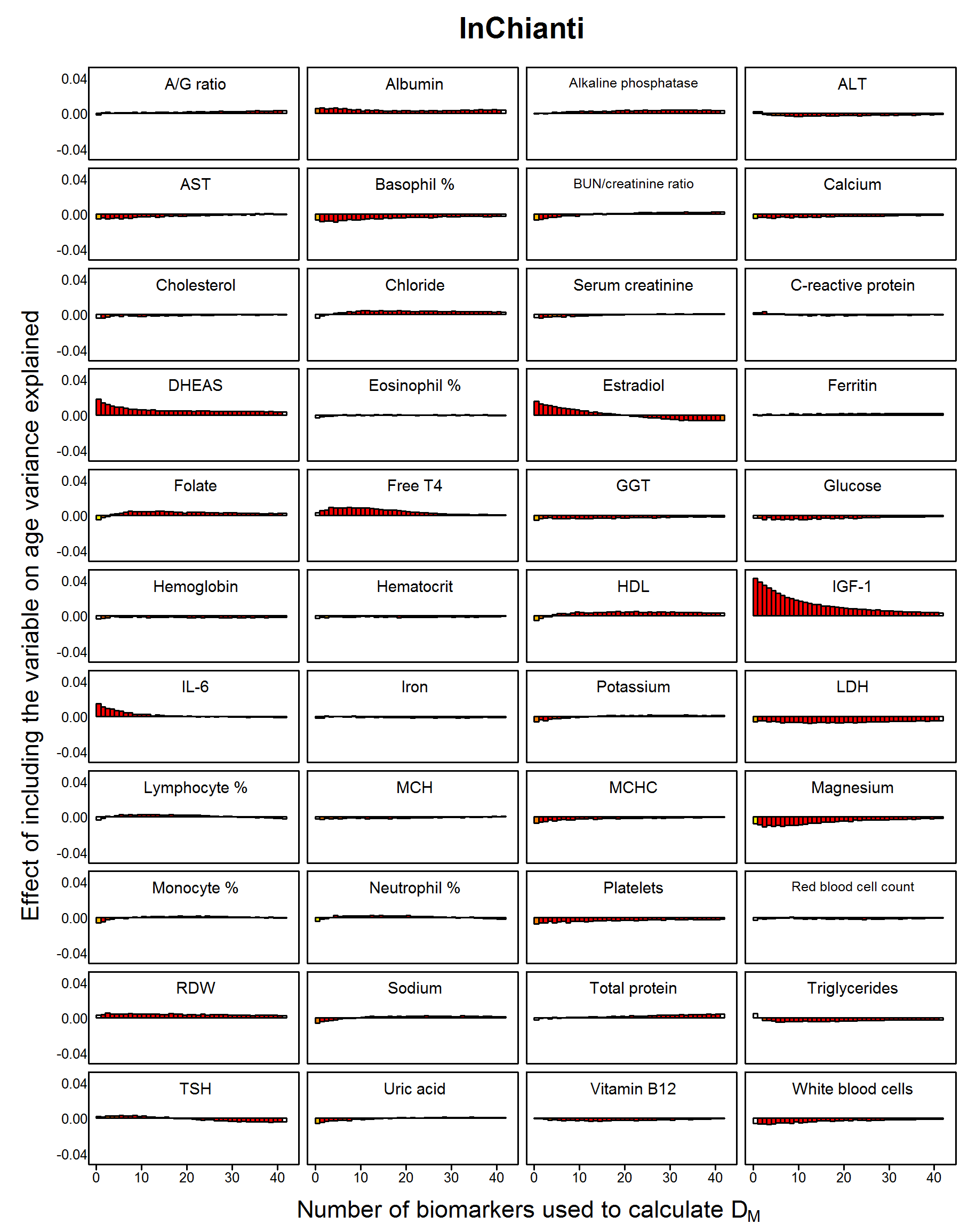

Supplement: S14 Fig — The X-axis represents the number of biomarkers per group (N bm), while the Y-axis reports the change in how much variance in age is predicted by D M with the inclusion of the given biomarker, based on a meta-regression of all the R-squareds calculated for individual quadratic regressions of age and D M. Colors indicate the magnitude of p-values, with darker red being more significant and white not significant. (TIF) [file pone.0122541.s016.tif]

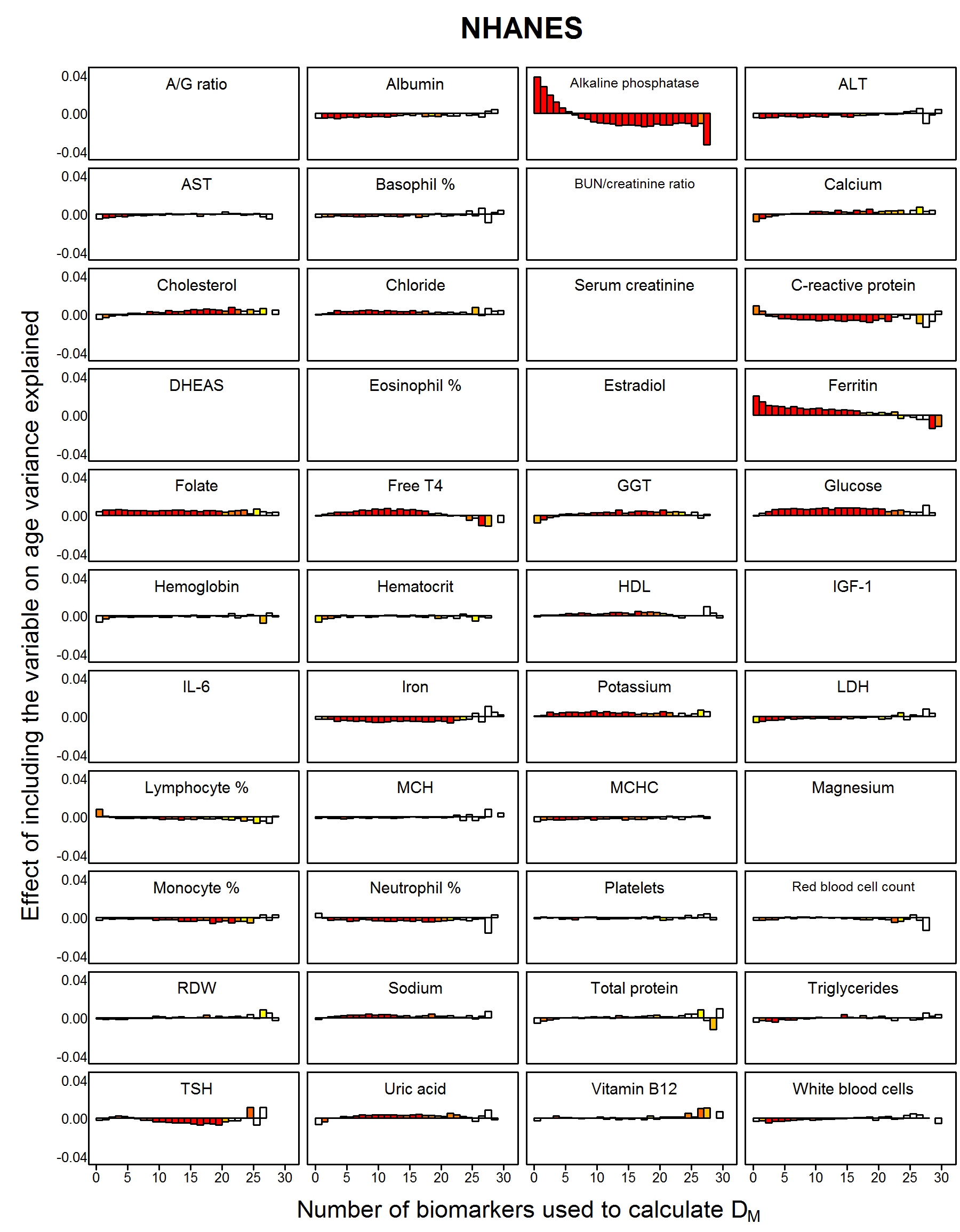

Supplement: S15 Fig — The X-axis represents the number of biomarkers per group (N bm), while the Y-axis reports the change in how much variance in age is predicted by D M with the inclusion of the given biomarker, based on a meta-regression of all the R-squareds calculated for individual quadratic regressions of age and D M. Colors indicate the magnitude of p-values, with darker red being more significant and white not significant. Empty panels are shown for biomarkers with no data for this particular data set (see text and Table 1 for details). (TIF) [file pone.0122541.s017.tif]

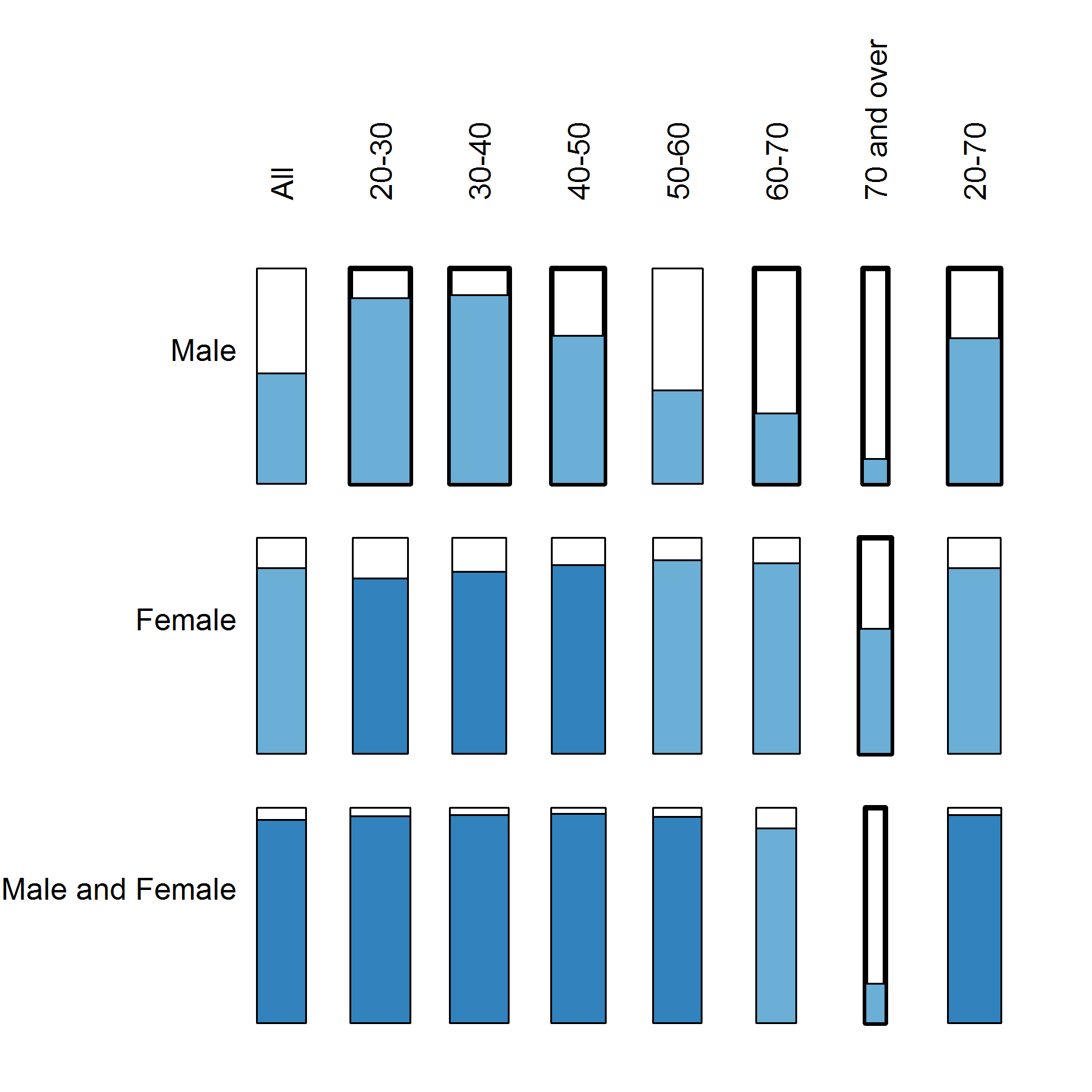

Supplement: S16 Fig — The study population represented here is the full NHANES data set stratified by sex as indicated, with RPs of differing age intervals (also NHANES). The width of the rectangle represents the average effect size (here the correlation between D M and age) among significant analyses, relative to the effect size of the rectangle in the leftmost column (entire study population as its own RP). The percentage of significant p-values is represented by the height of shading within the rectangle, the shading colour represents the direction of the effect (blue is a positive effect), and the hue represents the average p-value among the significant p-values, with darker hues indicating lower p-values. (TIF) [file pone.0122541.s018.tif]

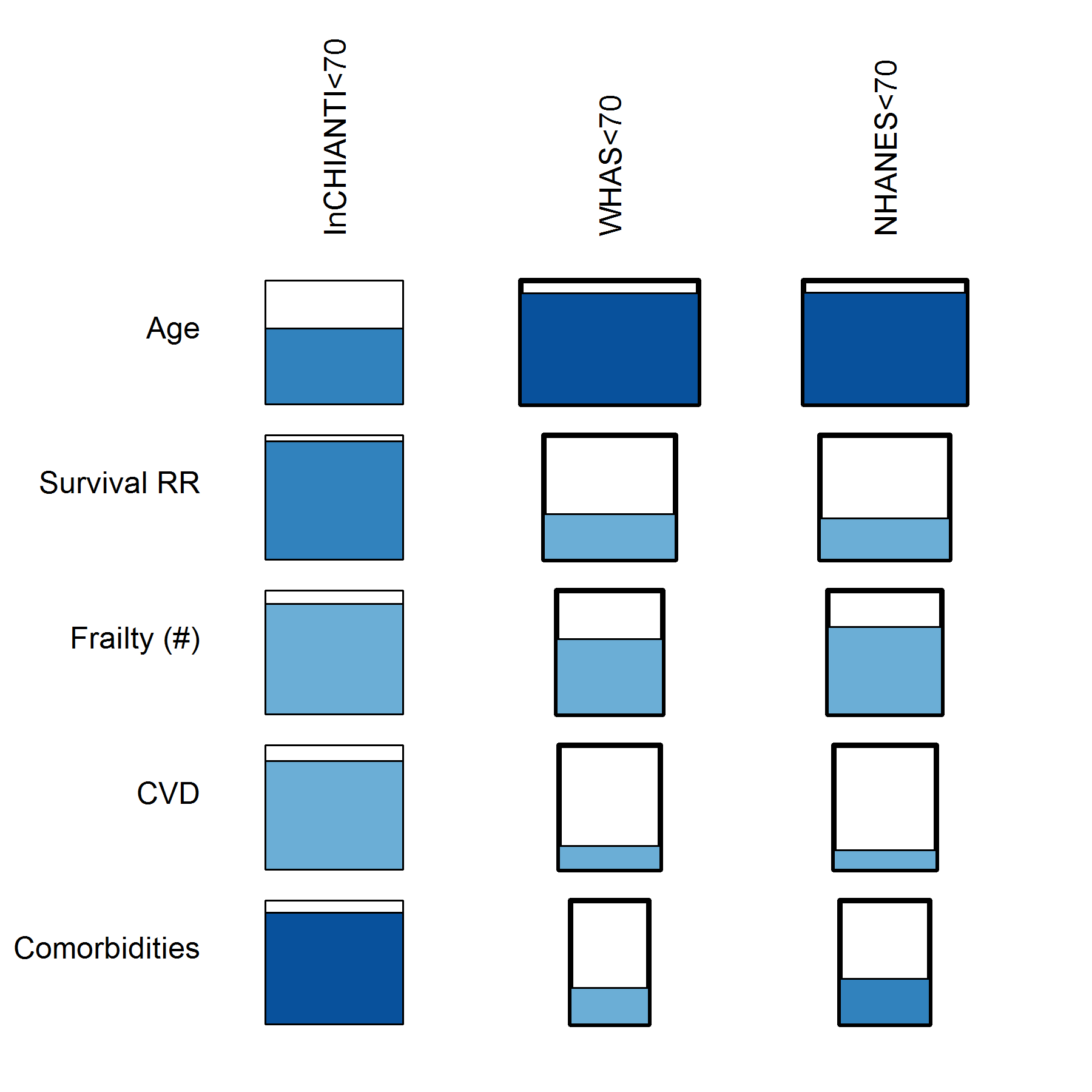

Supplement: S17 Fig — The study population represented here is the full InCHIANTI data set with RPs of young individuals from each of the three data sets. The width of the rectangle represents the average effect size among significant analyses, relative to the effect size of the rectangle in the leftmost column (entire study population as its own RP). The percentage of significant p-values is represented by the height of shading within the rectangle, the shading colour represents the direction of the effect (blue is a positive effect), and the hue represents the average p-value among the significant p-values, with darker hues indicating lower p-values. (TIF) [file pone.0122541.s019.tif]

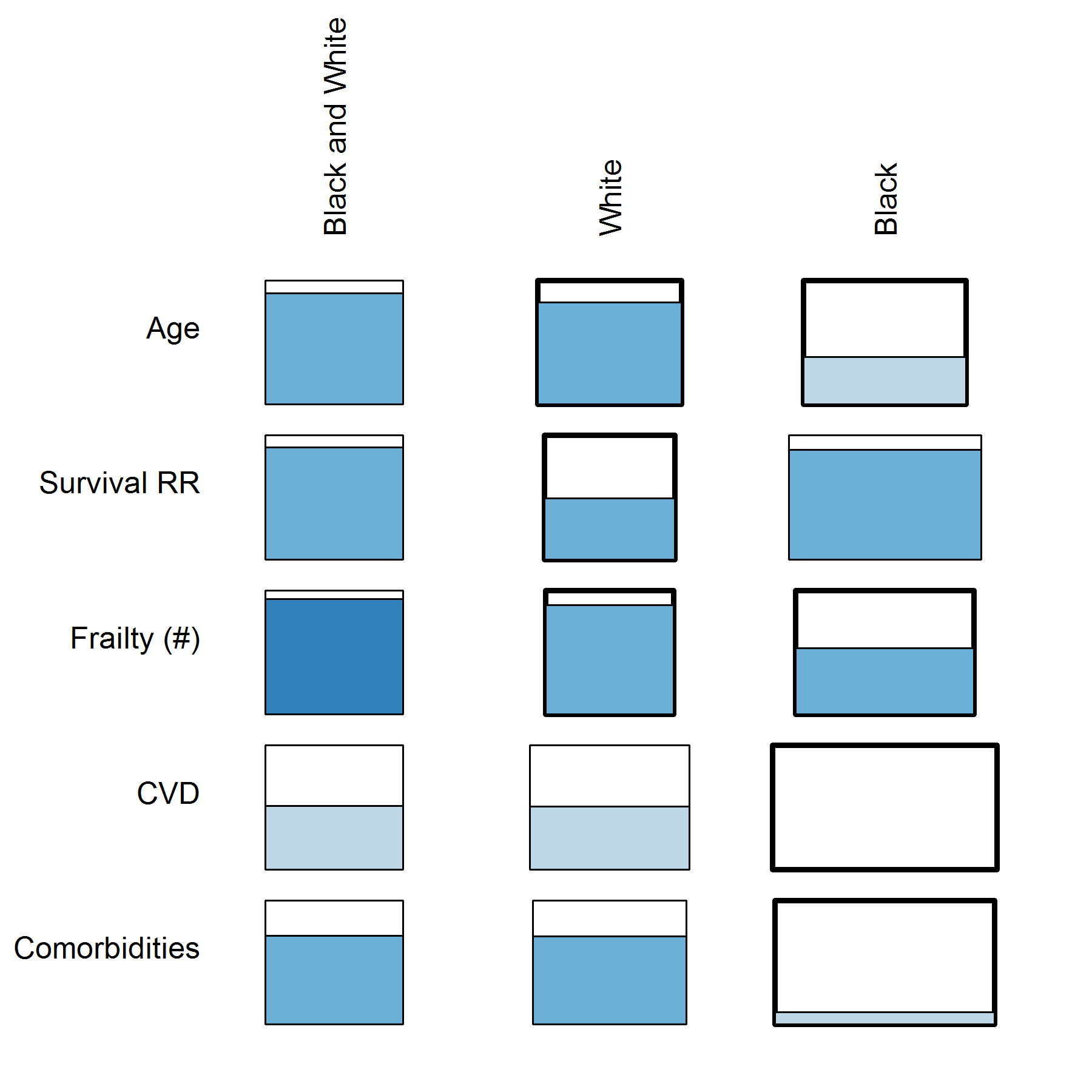

Supplement: S18 Fig — The study population represented here is the full WHAS data set using mixed, white-only, and black-only RPs (also WHAS). The width of the rectangle represents the average effect size among significant analyses, relative to the effect size of the rectangle in the leftmost column (entire study population as its own RP). The percentage of significant p-values is represented by the height of shading within the rectangle, the shading colour represents the direction of the effect (blue is a positive effect), and the hue represents the average p-value among the significant p-values, with darker hues indicating lower p-values. (TIF) [file pone.0122541.s020.tif]

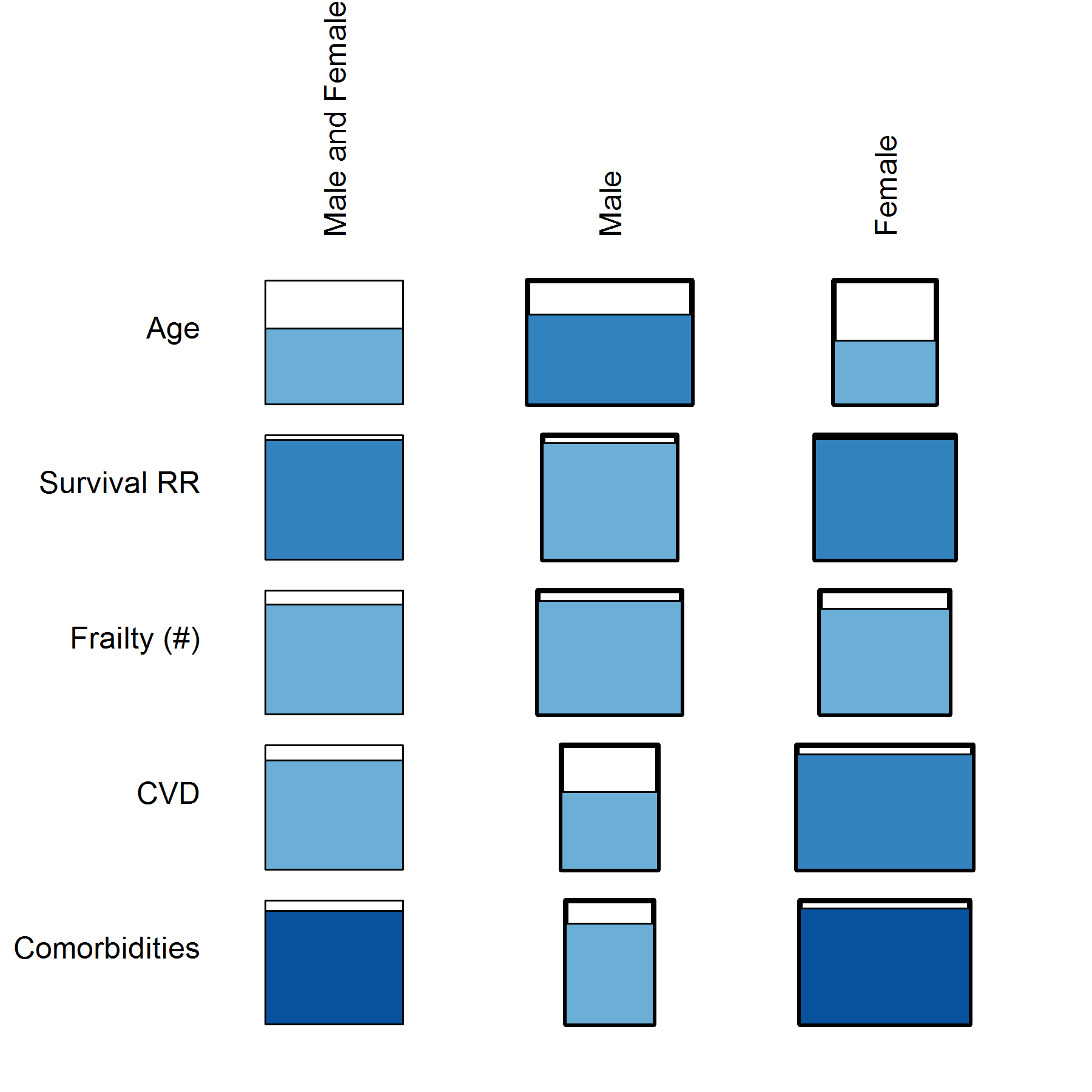

Supplement: S19 Fig — The study population represented here is the full InCHIANTI data set, varying the sex of the RP (also InCHIANTI). The width of the rectangle represents the average effect size among significant analyses, relative to the effect size of the rectangle in the leftmost column (entire study population as its own RP). The percentage of significant p-values is represented by the height of shading within the rectangle, the shading colour represents the direction of the effect (blue is a positive effect), and the hue represents the average p-value among the significant p-values, with darker hues indicating lower p-values. (TIF) [file pone.0122541.s021.tif]
